# Resolution of impaired multisensory processing in autism and the cost of switching sensory modality

**DOI:** 10.1038/s42003-022-03519-1

**Published:** 2022-06-30

**Authors:** Michael J. Crosse, John J. Foxe, Katy Tarrit, Edward G. Freedman, Sophie Molholm

**Affiliations:** 1 The Cognitive Neurophysiology Laboratory, Department of Pediatrics, Albert Einstein College of Medicine, Bronx, NY USA; 2 The Dominick P. Purpura Department of Neuroscience, Rose F. Kennedy Intellectual and Developmental Disabilities Research Center, Albert Einstein College of Medicine, Bronx, NY USA; 3 Trinity Centre for Biomedical Engineering, Department of Mechanical, Manufacturing & Biomedical Engineering, Trinity College Dublin, Dublin, Ireland; 4 The Cognitive Neurophysiology Laboratory, Del Monte Institute for Neuroscience, Department of Neuroscience, University of Rochester School of Medicine and Dentistry, Rochester, NY USA

**Keywords:** Autism spectrum disorders, Sensory processing, Human behaviour

## Abstract

Children with autism spectrum disorders (ASD) exhibit alterations in multisensory processing, which may contribute to the prevalence of social and communicative deficits in this population. Resolution of multisensory deficits has been observed in teenagers with ASD for complex, social speech stimuli; however, whether this resolution extends to more basic multisensory processing deficits remains unclear. Here, in a cohort of 364 participants we show using simple, non-social audiovisual stimuli that deficits in multisensory processing observed in high-functioning children and teenagers with ASD are not evident in adults with the disorder. Computational modelling indicated that multisensory processing transitions from a default state of competition to one of facilitation, and that this transition is delayed in ASD. Further analysis revealed group differences in how sensory channels are weighted, and how this is impacted by preceding cross-sensory inputs. Our findings indicate that there is a complex and dynamic interplay among the sensory systems that differs considerably in individuals with ASD.

## Abstract (web summary)

Crosse et al. study a cohort of 364 participants with autism spectrum disorders (ASD) and matched controls, and show that deficits in multisensory processing observed in high-functioning children and teenagers with ASD are not evident in adults with the disorder. Using computational modelling they go on to demonstrate that there is a delayed transition of multisensory processing from a default state of competition to one of facilitation in ASD, as well as differences in sensory weighting and the ability to switch between sensory modalities, which sheds light on the interplay among sensory systems that differ in ASD individuals.

## Introduction

Biological events tend to be multisensory, emanating or reflecting multiple forms of energy (e.g., photons, airborne vibrations, volatilised molecules, etc.). Humans have evolved a highly specialised set of sensory receptors that enable us to sample these different forms of energy concurrently, optimising how we perceive ecologically relevant information. For instance, processing redundant audiovisual cues often leads to faster response times (RTs) than processing the same information separately, a phenomenon known as the redundant signals effect (RSE)^1–3^. The RSE can be explained by parallel processing models such as the popular race model, which predicts that a response is triggered independently by the faster sensory channel^4^. However, the observed RSE typically exceeds the benefit predicted by mere statistical facilitation^5^. This multisensory effect has been demonstrated using bisensory detection tasks for several decades and is widely interpreted as reflecting the speed-up in processing time due to multisensory integration^6–15^.

Whereas multisensory processing clearly influences how we perceive most biological events, particularly in instances when sensory evidence is ambiguous^16–19^, individuals with autism spectrum disorders (ASD) often do not benefit from the availability of multisensory information to the same extent as their neurotypical (NT) peers^20–27^. We and others have suggested that impaired multisensory processing in ASD contributes to some of the commonly associated phenotypes such as atypical responses to sensory stimulation, and may even have detrimental effects on higher-order cognitive processes such as social interaction and communication^28–34^.

Previous studies using audiovisual (AV) speech stimuli have demonstrated that multisensory processing deficits observed in high-functioning children with ASD are not evident in teenagers with the disorder^25,35^. In contrast, when performing a simple AV detection task with flashes and beeps, high-functioning teenagers with ASD failed to show the same levels of multisensory gain as their NT peers^36^. Recent theoretical^32^ and computational^37^ perspectives have suggested that the constant exposure to AV speech during maturation may serve to train multisensory speech function, leading to resolution of AV speech deficits in ASD at an earlier age. However, the empirical question remains as to whether the resolution of multisensory deficits in ASD is specific to complex, social stimuli, or if instead it generalizes to more basic deficits in multisensory processing. This has obvious implications for understanding the basis of impairments in higher-order cognitive processes in ASD (e.g., social communication), as well as for neurobiological theories of ASD. Here, using the same bisensory detection task^36,38^, we tested the hypothesis that basic multisensory deficits observed in high-functioning children and teenagers with ASD would not be evident in adults with the disorder.

Electrophysiological studies in the cat superior colliculus have shown that the ability of neurons to integrate multisensory inputs is not present at birth^39,40^, but rather emerges and matures in the immature nervous system with exposure to multisensory experiences^41–44^. Computational modelling suggests that multisensory signals interact by default in a competitive manner, inhibiting effective processing of such stimuli^45^. Considerable postnatal exposure to multisensory cues is thought to strengthen excitatory cross-sensory projections, promoting processing of a facilitative nature^46–48^. While numerous developmental studies in humans have reported reduced multisensory ability in young children^49,50^, there is little empirical evidence of such competitive multisensory processing other than that reported in adults^51–53^. Here, we used a computational modelling framework to directly test whether behaviour in children reflected multisensory processing of a competitive or facilitative nature. The type of information processing was determined by how accurately hypothesis-driven models of multisensory behaviour could predict empirical multisensory benefits^15,54^. The same modelling approach was used to quantify age-related changes in sensory dominance and any potential group differences therein. We expected that such inherent sensory weighting would have a greater impact on multisensory processing of a competitive nature.

Multisensory experiments typically require participants to sequentially process different sensory stimuli in quick succession, and can thus be thought of as a task-switching paradigm. When switching from one modality to another, average response times are slower on trials preceded by a different sensory modality (switch trials) compared to trials preceded by the same modality (repeat trials)^55–57^. These so-called modality switch effects (MSEs) are inherent to any bisensory detection task that uses an intermixed stimulus presentation design^58,59^ and have been shown to systematically contribute to the RSE because they are typically larger on unisensory trials than on multisensory trials^60–62^. Moreover, data suggest that children with high-functioning ASD incur a greater cost when switching from auditory to visual stimuli than their NT peers^63^. We therefore examined potential group differences in MSEs and quantified their contribution to the RSE. Using a computational framework^64^, we modelled the trial-to-trial dependency between RTs on different sensory channels to gain deeper insight into how this process could be linked to inhibitory cross-sensory switching effects^53,65^. We discuss the implications of task-switching on the interpretation of the RSE, and how the interplay between multisensory integration and task-switching may contribute differentially as a function of age in NT and ASD individuals.

## Results

### Redundant signals effect

**Fig. 1:**
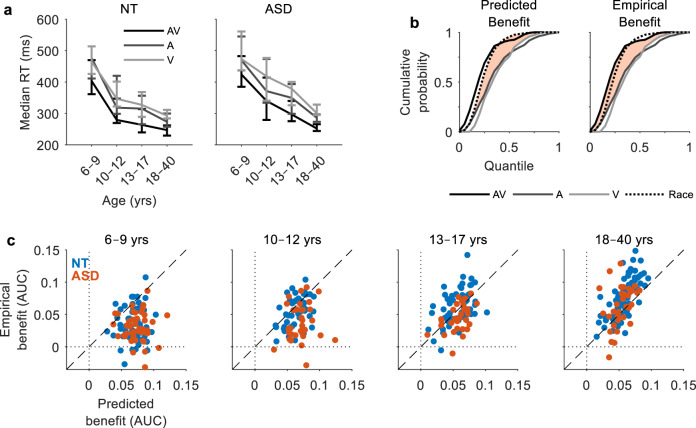
Reaction times and multisensory benefits. **a** Group median RTs for NT (left panel) and ASD (right panel) individuals as a function of age group. Error bars indicate 95% CIs (bootstrapped). **b** RT cumulative probability for each of the three stimulus conditions and the race model (Eq. 1). Predicted benefits (left panel) are quantified by the area between the CDFs of the race model and the faster of the unisensory conditions (Eq. 3). Empirical benefits (right panel) are quantified by the area between the CDFs of the multisensory condition and the faster of the unisensory conditions (Eq. 4). Data from an example NT adult participant. **c** Predicted benefits versus empirical benefits by age group. Each datapoint represents the area under the curve (AUC) for an individual participant (blue = NT, red = ASD).

In all, 225 neurotypical (NT) individuals (age range: 6–36 years; 115 females) and 139 individuals with a diagnosis of ASD (age range: 6–39 years; 34 females) performed a speeded bisensory detection task^36,38^ in which they were required to respond as fast as possible with a button press to randomly alternating auditory (A; 1 kHz tone), visual (V; red disk) and audiovisual (AV; disk/tone pair) stimuli, each with a duration of 60 ms and an interstimulus interval (ISI) of 1000–3000 ms (see Supplementary Fig. 1). To examine potential age-related effects, age was treated either as a continuous variable, or participants were separated into four cross-sectional age groups: 6–9, 10–12, 13–17 and 18–40 years (see Table 1 for group sample sizes and demographics). A linear mixed-effects analysis was used to examine the effects of group, age, and stimulus condition on response times (*R*^2^_adj_ = 0.53). ISI, preceding modality and subject were included as random factors, and the slopes of the latter 2 were adjusted for condition^66^ (see Methods section for further details). Participants with ASD responded slower to stimuli than their NT peers (*β* = 34.45, SE = 11.59, *p* = 0.003; Fig. 1a). There was an effect of age, with older participants responding faster than younger participants (*β* = −8.98, SE = 0.79, *p* = 1 × 10^−29^). Responses to multisensory stimuli were faster than those to both audio (*β* = 53.89, SE = 10.53, *p* = 3 × 10^−7^) and visual (*β* = 69.64, SE = 6.49, *p* = 8 × 10^−27^) stimuli, indicating the presence of an RSE. However, there was an interaction between age and RSE (RSE_A_: *β* = −0.58, SE = 0.21, *p* = 0.005; RSE_V_: *β* = −0.56, SE = 0.18, *p* = 0.002).

**Table 1:** Demographic characteristics of participant populations.

	NT				ASD			
	6–9 years	10–12 years	13–17 years	18–40 years	6–9 years	10–12 years	13–17 years	18–40 years
*n*	51	46	54	74	44	33	29	33
*n*_female_	27	26	24	38	7	6	8	13
*n*_IQ_	45	43	48	10	44	33	29	29
Age	8.1 (1.2)	11.5 (1.0)	15.0 (1.3)	25.3 (3.6)	8.1 (1.0)	11.4 (0.7)	14.7 (1.5)	24.8 (5.0)
*F*_1_ score	0.90 (0.07)	0.93 (0.06)	0.95 (0.04)	0.97 (0.02)	0.85 (0.08)	0.88 (0.08)	0.93 (0.06)	0.95 (0.04)
FA rate	0.09 (0.07)	0.06 (0.05)	0.04 (0.04)	0.02 (0.01)	0.16 (0.14)	0.13 (0.08)	0.07 (0.06)	0.04 (0.03)
Misses	0.11 (0.09)	0.07 (0.07)	0.04 (0.05)	0.03 (0.02)	0.15 (0.09)	0.12 (0.09)	0.07 (0.06)	0.05 (0.06)
PIQ	106.1 (13.0)	109.7 (10.7)	104.9 (13.3)	109.9 (12.3)	106.2 (17.1)	106.6 (16.2)	108.2 (13.3)	107.8 (14.2)
VIQ	113.0 (10.6)	111.8 (13.0)	113.1 (12.8)	115.1 (16.1)	97.3 (19.8)	99.4 (19.1)	100.0 (19.1)	104.0 (18.7)
FSIQ	111.4 (11.5)	112.2 (11.7)	110.1 (12.5)	114.5 (14.0)	101.7 (17.5)	102.8 (17.4)	104.3 (14.0)	106.2 (16.7)
ADOS	–	–	–	–	7.3 (2.3)	8.1 (1.0)	6.9 (3.3)	–

*PIQ* performance IQ, *VIQ* verbal IQ, *FSIQ* full-scale IQ (assessed using the WASI).

*n*_female_ indicates the number of female participants in the respective age groups and *n*_IQ_ indicates the number of participants for whom IQ scores were obtained. The number of participants for whom ADOS scores were obtained is 31, 19, 7 respectively.

*F*_1_ scores indicate participants’ detection accuracy and FA rate indicates participant’s false alarm rate as a proportion of the total trials. Values shown indicate the group mean with standard deviation shown in parentheses.

To examine the RSE further, we measured the proportion of RTs that were faster on multisensory trials relative to the faster of the unisensory conditions, i.e., multisensory benefit^54^. For this, RT distributions were converted to cumulative distribution functions (CDFs) to enable analysis of the whole RT distribution^67,68^. Empirical benefits were quantified by the area between the CDFs of the multisensory condition and the faster of the unisensory conditions (Eq. 4; Fig. 1b, right). Analogously, predicted benefits were quantified by the area between the CDFs of the race model and the faster of the unisensory conditions (Eq. 3; Fig. 1b, left)^54^. Here, we used Raab’s independent race model^4^, which assumes statistical independence between sensory channels (see Methods for further details). A linear regression model was constructed to quantify the effects of group and age on predicted (*R*^2^_adj_ = 0.09, BF_01_ = 9 × 10^−6^) and empirical (*R*^2^_adj_ = 0.25, BF_01_ = 2 × 10^−21^) benefits. Predicted benefits decreased as a function of age (*β* = −0.001, SE = 0.0001, *p* = 2 × 10^−8^, BF_01_ = 3 × 10^−6^) and were not significantly different in NT and ASD individuals (*β* = 0.002, SE = 0.0019, *p* = 0.28, BF_01_ = 9.1). Conversely, empirical benefits increased with age (*β* = 0.002, SE = 0.0002, *p* = 1 × 10^−19^, BF_01_ = 3 × 10^−17^) and were smaller in individuals with ASD (*β* = −0.013, SE = 0.003, *p* = 1 × 10^−5^, BF_01_ = 1 × 10^−3^). This suggests that the race model over-predicts empirical benefits for younger individuals and under-predicts them for older individuals (see Fig. 1c). Moreover, the race model does not predict the group differences observed in empirical benefits, suggesting a deficit specific to multisensory processing.

### Testing the race model

**Fig. 2:**
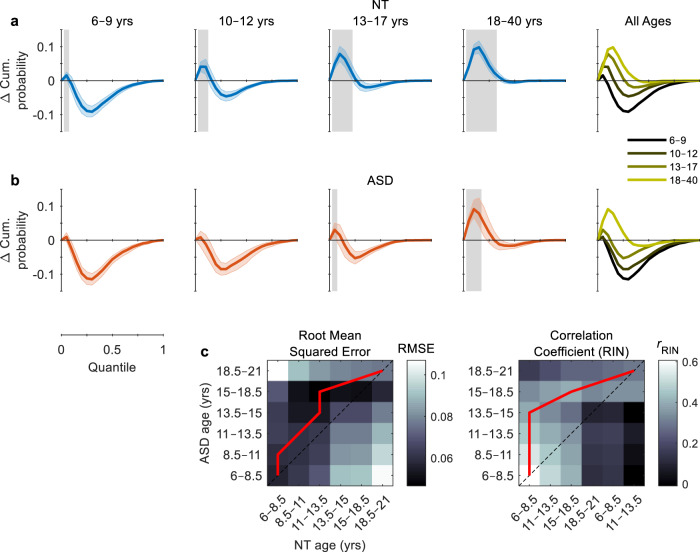
Testing the race model. **a**, **b** Multisensory facilitation was quantified by the difference between the CDFs of the multisensory condition and the independent race model. Positive deviations reflect the proportion of multisensory RTs that were faster than predicted by the race model. Gray shaded regions indicate significant positive deviations (*p* < 0.05, right-tailed permutation tests, *t*_max_ corrected). Coloured error bounds indicate 95% CIs (bootstrapped). **c** Root mean squared error (left panel) and RIN-transformed Pearson correlation coefficient (right panel) between the difference functions for NT and ASD participants of different ages (range: 6–21 years, increment: 2.5 years). Red lines indicate the minimum (left panel) and maximum (right panel) values of each row (i.e., the NT groups that were most similar to each ASD group). Divergence of the red line above the dotted midline indicates a potential delay in age-related changes in ASD.

To test whether the speed-up due to multisensory processing exceeded statistical facilitation, we quantified the proportion of audiovisual RTs that deviated from the predictions of Raab’s independent race model at every RT quantile. Positive deviations indicate multisensory facilitation and were detected using right-tailed permutation tests with *t*_max_ correction^69^. NT participants showed evidence of facilitation at one or more quantiles in every age group, the number of quantiles increasing as a function of age (*p* < 0.05, shaded area, Fig. 2a). The percentage of participants that exhibited facilitation is illustrated in Supplementary Fig. 2. Individuals with ASD showed no evidence of facilitation between the ages of 6–12 years (Fig. 2b). However, evidence of facilitation emerges in adolescence (first quantile) and becomes more evident in adulthood (first 3 quantiles; see Supplementary Table 2 for test statistics).

To compare deviations from the race model between NT and ASD individuals of different ages, we computed the root-mean-square error (RMSE) and correlation coefficient between each participant’s difference function and that of every other participant. Prior to assessing the Pearson correlation, a rank-based inverse normal (RIN) transformation was applied to the data^70^. Participants were split into 10 age bins separated by 2.5 years between the ages of 6–21 years. Similarity matrices containing RMSE and correlation values were obtained by averaging over the values within each age bin (Fig. 2c). The red lines indicate the NT groups that were most similar to each ASD group, and its divergence above the dotted midline suggests that multisensory behaviour in individuals with ASD corresponded more closely to that of younger NT individuals (i.e., a potential delay in age-related changes). Convergence of the red lines towards the midline in 18.5–21-year-olds suggests that this delay may resolve in adulthood, in line with our original hypothesis. This absence of multisensory deficits in adulthood is further examined in the following section.

### Resolution of multisensory deficits in ASD

**Fig. 3:**
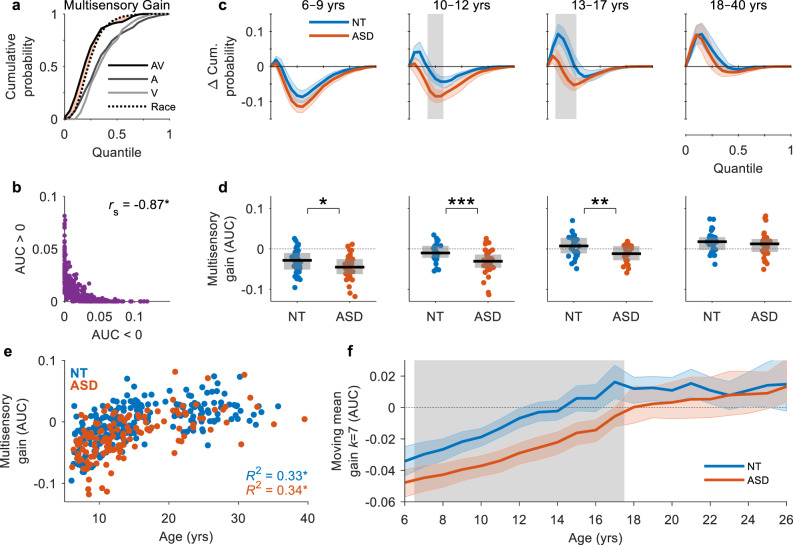
Resolution of multisensory deficits in ASD. **a** RT cumulative probability for each of the three stimulus conditions and the race model. Multisensory gain is quantified by the area between the CDFs of the multisensory condition and the race model (Eq. 5). Data from an example NT adult participant. **b** The area under the curve (AUC) below zero is negatively correlated with the AUC above zero, providing information about participants that do not exhibit facilitation. **c** Race model violation for ASD (red trace) and sex, age, and IQ-matched NT (blue trace) participants by age group. Coloured error bounds indicate 95% CIs (bootstrapped). Gray shaded regions indicate significant group differences (*p* < 0.05, two-tailed permutation tests, *t*_max_ corrected). **d** Multisensory gain by age group. Boxplots indicate the median value (black line) and interquartile range (grey box). Each datapoint represents the AUC of an individual participant (blue = NT, red = ASD). Brackets indicate unpaired statistical comparisons (*p < 0.05, **p < 0.01, ***p < 0.001, two-tailed permutation tests, FDR corrected). **e** Multisensory gain as a function of age for NT (left) and ASD (right) individuals. Each datapoint represents the AUC of an individual participant. **f** Mean multisensory gain calculated with a moving window *k* of 7 years in increments of 1 year from 6 to 26 years for NT (blue trace) and ASD (red trace) participants. Participants were sex, age and IQ-matched within each 7-year window. Coloured error bounds indicate 95% CIs (bootstrapped). Gray shaded regions indicate significant group differences (*p* < 0.05, two-tailed permutation tests, FDR corrected).

To examine this potential delay in age-related changes in ASD, we constructed a linear model to evaluate the effects of group and age on multisensory gain (*R*^2^_adj_ = 0.39, BF_01_ = 1 × 10^−35^). Multisensory gain was quantified by the net proportion of multisensory RTs that were faster than the independent race model (Eq. 5; Fig. 3a). Multisensory gain exhibited a moderate increase as a function of age (*β* = 0.0025, SE = 0.0002, *p* = 4 × 10^−21^, BF_01_ = 0.98) but was significantly reduced in participants with ASD compared to NT individuals (*β* = −0.023, SE = 0.007, *p* = 0.001, BF_01_ = 6 × 10^−5^). The absence of an interaction between group and age suggests that this age-related effect was present in both groups (*β* = 0.0005, SE = 0.0004, *p* = 0.22, BF_01_ = 8.84). Group comparisons were conducted within each of the four age groups. For this, NT participants were sex, age and IQ-matched to each of the ASD participants and difference functions were compared at every quantile using two-tailed (unpaired) permutation tests (see Methods section for further details). Group differences were observed in the 10–12-year-olds at quantiles 4–6, and in the 13–17-year-olds at quantiles 2–5 (*p* < 0.05, shaded area, Fig. 3c). To determine whether this multisensory deficit in ASD can be said to resolve in adulthood, a Bayes factor analysis was used to provide evidence in favour of the absence of any group differences based on the net multisensory gain (Fig. 3d). Group differences were observed in participants aged 6–9 years (*t*_(86)_ = 2.37, *p* = 0.021, Hedge’s *g* = 0.50, 95CI [0.09, 0.94], BF_01_ = 0.47), 10–12 years (*t*_(64)_ = 3.48, *p* = 0.001, Hedge’s *g* = 0.85, 95CI [0.40, 1.36], BF_01_ = 0.03) and 13–17 years (*t*_(56)_ = 3.13, *p* = 0.003, Hedge’s *g* = 0.81, 95CI [0.33, 1.37], BF_01_ = 0.08), but there was no evidence to suggest a group difference in participants aged 18–40 years (*t*_(64)_ = 1.02, *p* = 0.310, Hedge’s *g* = 0.25, 95CI [−0.23, 0.77], BF_01_ = 3.34).

To examine these age-related changes in greater detail, we treated age as a continuous variable (Fig. 3e). Age was highly predictive of multisensory gain between 6–17 years (NT: *R*^2^ = 0.34, *p* = 2 × 10^−5^, BF_01_ = 7 × 10^−13^; ASD: *R*^2^ = 0.21, *p* = 2 × 10^−5^, BF_01_ = 8 × 10^−5^) but not between 18–40 years (NT: *R*^2^ = 0.005, *p* = 0.56, BF_01_ = 9.22; ASD: *R*^2^ = 0.015, *p* = 0.5, BF_01_ = 5.92), suggesting that progression of this process ceases in adulthood. To characterise the trajectory of multisensory processing as a function of age more precisely, we calculated the mean multisensory gain with a moving window *k* of 7 years in increments of 1 year between the ages of 6 and 26 years (Fig. 3f). Controls were sex, age and IQ-matched to ASD individuals within each 7-year window and compared using two-tailed permutation tests (FDR corrected) and Bayes factor analyses. In NT participants, multisensory gain increased steadily between the ages of 6–17 years. In individuals with ASD, the rate of increase was slower and the average gain was significantly lower than that of their NT peers between 6–17 years (*p* < 0.05, shaded area, Fig. 3f). In adults aged 18 years and over, there was no evidence to suggest any significant group differences (*p* > 0.05). Moreover, our Bayes factor analysis provided evidence in favour of the absence of an effect between the ages of 22–26 years (BF_01_ > 3; see Supplementary Fig. 3), suggesting that this deficit resolves in adulthood as originally hypothesised.

### Modelling multisensory behaviour in ASD

**Fig. 4:**
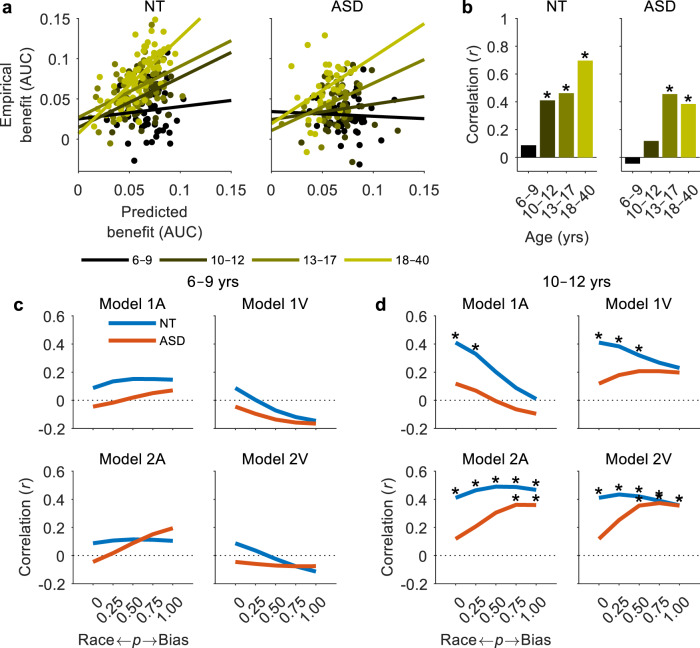
Modelling multisensory behaviour. **a** Predicted benefits versus empirical benefits for NT (left panel) and ASD (right panel) participants. Each datapoint represents the AUC of an individual participant and age group is indicated by colour. Solid lines represent linear fits to the data by age group. **b** Pearson correlation coefficient (*r*) of the regression fits in **a**. Asterisks indicate significant correlations (*p* < 0.05, two-tailed permutation tests). **c**, **d** Hypothetical models of multisensory competition were tested. Model 1A was biased towards the auditory modality and Model 1V towards the visual modality (Eq. 6). Model 2A was biased towards the preceding modality and the A modality when preceded by an AV trial, and Model 2V was biased towards the preceding modality and the V modality when preceded by an AV trial (Eq. 7). The probability *p* of multisensory processing being facilitative (race model) or competitive (bias model) was parametrically varied between 0 and 1 in increments of 0.25 (Eq. 8). The ability of the models to predict empirical benefits was assessed within each age group based on the Pearson correlation coefficient. Data presented are the two younger age groups. See Supplementary Fig. 4 for the two older age groups.

Raab’s independent race model has been shown to provide strong predictions of the RSE in healthy adults, suggesting that the basic underlying neural architecture may follow that of the race model^15,54,64^. This is also consistent with the fact that the logic operations of the race model (i.e., logic OR gate) exactly match the tasks demands of a bisensory detection task (i.e., respond to the presence of a signal on channel A *or* channel V)^59^. To assess whether the independent race model could provide similar predictions of multisensory behaviour in children and individuals with ASD, one-way ANCOVAs were used to measure the correlation between predicted and empirical benefits in each age group by treating age group as a partialled out categorical variable^71^. Predicted benefits were correlated with empirical benefits in NT individuals (*F*_(1,217)_ = 62.88, *p* = 1 × 10^−13^, *R*^2^ = 0.23, BF_01_ = 1 × 10^−19^) and less so in individuals with ASD (*F*_(1,131)_ = 5.64, *p* = 0.019, *R*^2^ = 0.041, BF_01_ = 0.35), but an interaction between age group and predicted benefits in NT individuals suggested that this relationship was age-dependent (NT: *F*_(3,217)_ = 5.4, *p* = 0.0013, *R*^2^ = 0.07, BF_01_ = 0.97; ASD: *F*_(3,131)_ = 2.22, *p* = 0.088, *R*^2^ = 0.05, BF_01_ = 51.9). Figure 4a, b shows that the ability of the race model to predict empirical benefits increases dramatically as a function of age. While the race model predicted a significant proportion of the variance in the 18–40-year-olds (NT: *R*^2^ = 0.49, *p* = 2 × 10^−5^, BF_01_ = 6 × 10^−10^; ASD: *R*^2^ = 0.15, *p* = 0.028, BF_01_ = 0.66), it accounted for almost none of the variance in the 6–9-year-olds (NT: *R*^2^ = 0.008, *p* = 0.54, BF_01_ = 7.58; ASD: *R*^2^ = 0.002, *p* = 1.0, BF_01_ = 8.15).

In light of this result and recent findings, we wished to test the hypothesis that multisensory processing in early childhood is governed by a competition rather than facilitation^45^. While facilitation would likely follow the predictions of the race model, we hypothesized that competition would follow the predictions of the more dominant sensory modality. Thus, we tested the predictions of models that were biased towards either a specific modality (Models 1A and 1V; Eq. 6) or the previous modality (Models 2A and 2V; Eq. 7). The probability *p* of multisensory processing being facilitative (race model) or competitive (bias model) was parametrically varied between 0 and 1 in increments of 0.25 (Eq. 8; see Methods section for further details). In children with ASD aged 6–9 years, Model 2A was most accurate at predicting empirical benefits, suggesting that their responses were triggered by the previous modality with an auditory bias (Fig. 4c). In their NT peers, none of the bias models exceeded the performance of the race model considerably, but there was evidence in favour of an auditory bias once again. In ASD participants aged 10–12 years, Model 2V dramatically exceeded race model performance, suggesting that RTs were largely determined by the previous modality but this time with a visual bias (Fig. 4d). In their NT peers, there was no major improvement beyond the race model, but there was evidence of a visual bias as well. In teenagers and adults, none of the bias models outperformed the race model, suggesting that individuals with ASD transition from competition to facilitation during adolescence (Supplementary Fig. 4).

### Age-related changes in sensory dominance

**Fig. 5:**
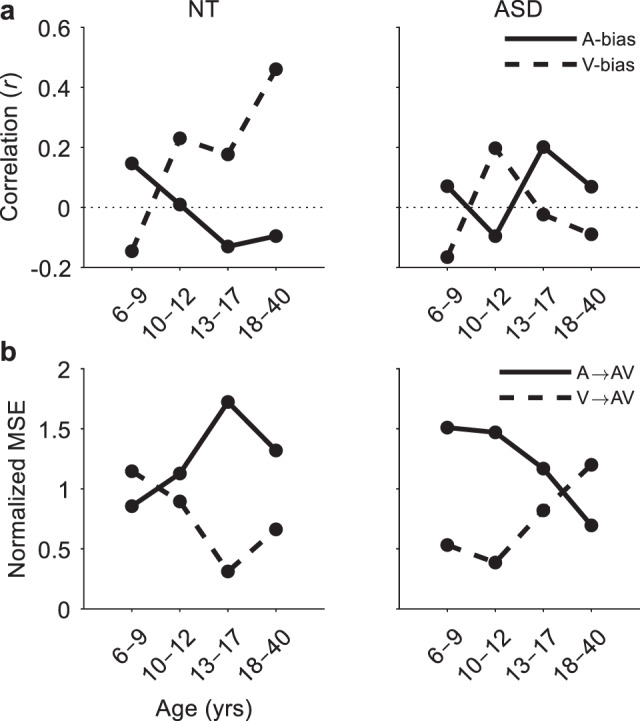
Age-related changes in sensory dominance. **a** Sensory dominance was examined by measuring the performance of Model 1A (auditory bias, solid trace) and Model 1V (visual bias, dotted trace) with the probability of a sensory-specific bias *p* set to 1 (Eq. 8). The ability of each model to predict empirical benefits was assessed within each age group based on the Pearson correlation coefficient. **b** Modality switch effects for AV trials separated by trials preceded by A-stimuli (solid trace) and V-stimuli (dotted trace). MSEs were quantified by the area between the CDFs of the switch and repeat trials and normalised by the grouped V/A→AV MSEs.

To further examine age-related patterns in sensory dominance, we tested Model 1A and Model 1V with the probability of a sensory-specific bias set to 1 (Eq. 8). Evaluating model performance as before, we noticed an auditory dominance in both groups at 6–9 years that shifted to a visual dominance by 10–12 years (Fig. 5a). In the NT group, this visual dominance appears to continue into adulthood in accordance with the well-known Colavita visual dominance effect^72^. However, in the ASD group, this sensory weighting appears to shift once again in adolescence, leading to an apparent auditory dominance in adulthood.

If such biases exist, one would expect to see larger switch costs on AV trials preceded by the less dominant modality than those preceded by the dominant modality. To test this hypothesis, we examined modality switch effects (MSEs) on AV trials, separating trials preceded by A and V trials (A→AV and V→AV, respectively). MSEs were quantified by deriving separate CDFs for switch and repeat trials and computing the area between them (Eq. 9; see Methods section for further details). Additionally, MSEs in each condition were normalized by that of the grouped condition (V/A→AV) to allow for meaningful comparison across age groups (this did not change the results qualitatively). Based on our modelling of sensory dominance, we expected to see greater MSEs on V→AV trials for NT children aged 6–9 years, and on A→AV trials for NT individuals aged 10 years and over. We expected something similar for ASD individuals with another shift in adolescence. The data in Fig. 5b suggest that, as predicted, MSEs were greater on V→AV trials for NT 6–9-year-olds and on A→AV trials for NT 10–40-year-olds. For ASD individuals, the data suggest the reverse, with greater MSEs on A→AV trials in children and teenagers (6–17 years) and on V→AV trials for adults (18–40 years).

### Reduced switch costs in ASD

**Fig. 6:**
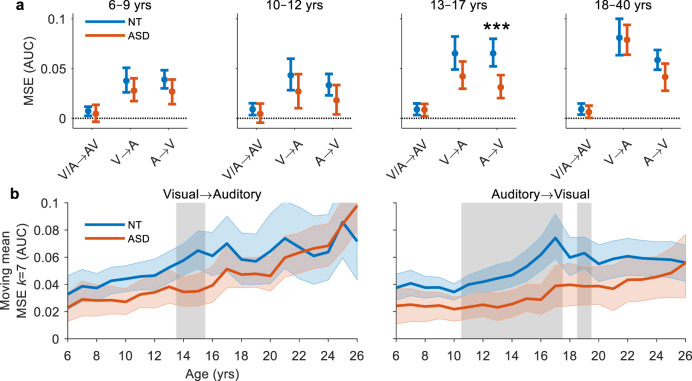
Modality switch effects. **a** Mean MSE for each condition by age group. MSEs were quantified by the area between the CDFs of the switch and repeat trials (Eq. 9). Error bars indicate 95% CIs (bootstrapped). Asterisks indicate significant group differences (*p* < 0.05, two-tailed permutation tests, *t*_max_ corrected). **b** Mean MSE for visual to auditory (left panel) and auditory to visual (right panel) switches calculated with a moving window *k* of 7 years in increments of 1 year from 6–24 years for NT (blue trace) and ASD (red trace) participants. Coloured error bounds indicate 95% CIs (bootstrapped). Gray shaded regions indicate significant group differences (*p* < 0.05, two-tailed permutation tests, FDR corrected).

To detect potential group differences in the behavioural cost of switching sensory modality, we modelled the effects of diagnosis, age, and condition on MSEs (*R*^2^_adj_ = 0.304, BF_01_ = 2 × 10^−80^). MSEs increased with age (*β* = 0.002, SE = 0.0002, *p* = 1 × 10^−24^, BF_01_ = 7 × 10^−21^) and, contrary to previous work^63^, were reduced in individuals with ASD compared to NT individuals (*β* = −0.012, SE = 0.002, *p* = 4 × 10^−7^, BF_01_ = 9 × 10^−5^). Compared to multisensory trials, MSEs were larger on both auditory trials (*β* = 0.048, SE = 0.003, *p* = 2 × 10^−59^, BF_01_ = 3 × 10^−59^) and visual trials (*β* = 0.037, SE = 0.003, *p* = 7 × 10^−38^, BF_01_ = 3 × 10^−59^). Follow-up permutation tests and Bayes factor analyses revealed that MSEs were only reduced in the adolescent ASD group when switching from auditory to visual stimuli (*t*_(56)_ = 3.63, *p* = 0.001, Hedge’s *g* = 0.94, 95CI [0.46, 1.52], BF_01_ = 0.02; Fig. 6a). A more detailed examination using a moving mean estimate of MSE showed that these group differences emerged between the ages of 11–17 years (*p* < 0.05, shaded area, FDR corrected; Fig. 6b, right) and were strongest at around 16 years (Hedge’s *g* = 0.93, 95CI [0.48, 1.42], BF_01_ = 0.01). For visual to auditory switches, MSEs were once again reduced in individuals with ASD between 14 and 16 years of age (Hedge’s *g* = 0.68, 95CI [0.27, 1.14], BF_01_ = 0.07). For effect size and Bayes factor analyses, see Supplementary Fig. 5.

Contrary to our results, a study by Williams, et al.^63^ found that individuals with ASD between the ages of 8–15 years exhibited a greater cost to switching from auditory to visual stimuli than their age-matched NT peers. To test this directly, we performed a left-tailed permutation test and Bayes factor analysis on a group of sex, age and IQ-matched participants between the ages of 8–15 years (*n* = 79 per group) and used a similar measure of MSE based on the mean RT. However, this analysis did not suggest that MSEs were greater in individuals with ASD (NT: 30.0 ± 27.4 ms, ASD: 19.7 ± 42.2 ms; *t*_(156)_ = 1.82, *p* = 0.964, Hedge’s *g* = 0.29, 95CI [−0.01, 0.60], BF_01_ = 23.58), confirming that the inconsistency between our studies was not a consequence of how MSE was quantified. The only remaining difference between our studies was that Williams, et al.^63^ used longer ISIs (3–5 s vs. 1–3 s). Thus, we repeated the test, but limited RTs to trials with preceding ISIs between 2.5–3 s. Once again, there was no evidence to suggest that MSEs were greater in individuals with ASD (*t*_(156)_ = 1.30, *p* = 0.905, Hedge’s *g* = 0.21, 95CI [−0.10, 0.51], BF_01_ = 18.43). However, limiting the analysis to longer ISIs did result in a reduction in mean MSE in both NT individuals (18.5 ± 43.5 ms, *t*_(78)_ = 3.01, *p* = 0.008, Hedge’s *g* = 0.32, 95CI [0.11, 0.58], BF_01_ = 0.16) and individuals with ASD (7.5 ± 60.7 ms, *t*_(78)_ = 1.95, *p* = 0.102, Hedge’s *g* = 0.23, 95CI [0.00, 0.48], BF_01_ = 1.79), suggesting invocation of disparate processing underlying MSEs at shorter versus longer ISIs.

### Modelling channel dependency and RT variability

**Fig. 7:**
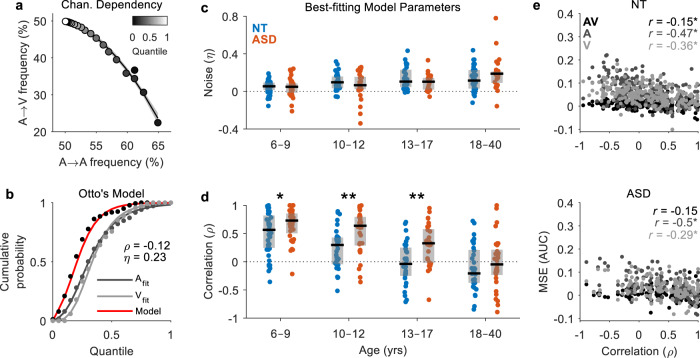
Modelling channel dependency and RT variability. **a** Frequency of visual and auditory trials preceded by auditory trials in each quantile (i.e., switch vs. repeat trials). Quantiles are indicated by a grayscale, graduating from black (fastest quantile) to white (slowest quantile). Example data averaged over all NT adult participants. **b** CDFs were fit to the unisensory RT data and used to predict empirical multisensory RT data via Otto’s context variant race model^64^. Free parameters *ρ* and *η* account for the correlation between RTs on different channels and increased RT variability or noise, respectively. Data from an example NT adult participant. **c**, **d** Best-fitting model parameters *ρ* and *η* by diagnosis and age group. Boxplots indicate the median value (black line) and interquartile range (grey box). Each datapoint represents an individual participant (blue = NT, red = ASD). **e** Modality switch effect (MSE) as a function of model parameter *ρ* for NT (top panel) and ASD (bottom panel) participants. Each datapoint represents an individual participant (black = AV, dark grey = A, light grey = V).

To gain deeper insights into the factors that shape multisensory facilitation and switch costs, we adopted a computational modelling framework developed by Otto and Mamassian^64^. This framework extends Raab’s independent race model such that it includes 2 additional free parameters, *η* and *ρ* (Fig. 7b), that enable a more accurate fit to empirical multisensory RTs (see Methods for further details). The first parameter *η* accounts for the additional variability or noise typically observed in empirical multisensory RTs relative to that predicted by Raab’s race model; this increase could be due to the pooling of neuronal activity (and variability) during multisensory processing. The second parameter *ρ* allows us to quantify the trial-to-trial correlation between RTs on different sensory channels; this correlation typically presents as a negative channel dependency for healthy adults because neural processing on one channel likely happens at the expense of processing on the other^5,64^. We can visualise this negative dependency by plotting the average frequency of switch versus repeat trials per quantile in our NT adult group (see Fig. 7a). We hypothesized that the increase in RT variability would be larger for individuals with higher multisensory gain, and that channel dependency would be lower or more negatively correlated for individuals with greater MSEs. The best-fitting estimates of the noise parameter *η* increased with age (*β* = 0.005, SE = 0.0008, *p* = 2 × 10^−10^) but there was no evidence to suggest a group difference (*β* = −0.007, SE = 0.012, *p* = 0.58; *R*^2^_adj_ = 0.105; Fig. 7c). The best-fitting estimates of the correlation parameter *ρ* decreased with age (*β* = −0.037, SE = 0.003, *p* = 5 × 10^−33^) and were more positive-going in ASD (*β* = 0.22, SE = 0.04, *p* = 2 × 10^−7^; *R*^2^_adj_ = 0.382; Fig. 7d). Follow-up tests revealed group differences in participants aged 6–9 years (*t*_(86)_ = 2.32, *p* = 0.021, Hedge’s *g* = 0.49, 95CI [0.08, 0.93], BF_01_ = 0.44), 10–12 years (*t*_(64)_ = 3.27, *p* = 0.002, Hedge’s *g* = 0.80, 95CI [0.31, 1.41], BF_01_ = 0.05) and 13–17 years (*t*_(56)_ = 3.20, *p* = 0.002, Hedge’s *g* = 0.83, 95CI [0.32, 1.44], BF_01_ = 0.06). As predicted, channel dependency was more negative for participants that exhibited larger MSEs in both NT individuals (*r*_AV_ = −0.15, *p* = 0.02; *r*_A_ = −0.47, *p* = 2 × 10^−4^; *r*_V_ = −0.36, *p* = 2 × 10^−4^) and individuals with ASD (*r*_AV_ = −0.15, *p* = 0.02; *r*_A_ = −0.5, *p* = 2 × 10^−4^; *r*_V_ = −0.29, *p* = 2 × 10^−4^; Fig. 7e).

### Linking sequential and multisensory effects

**Fig. 8:**
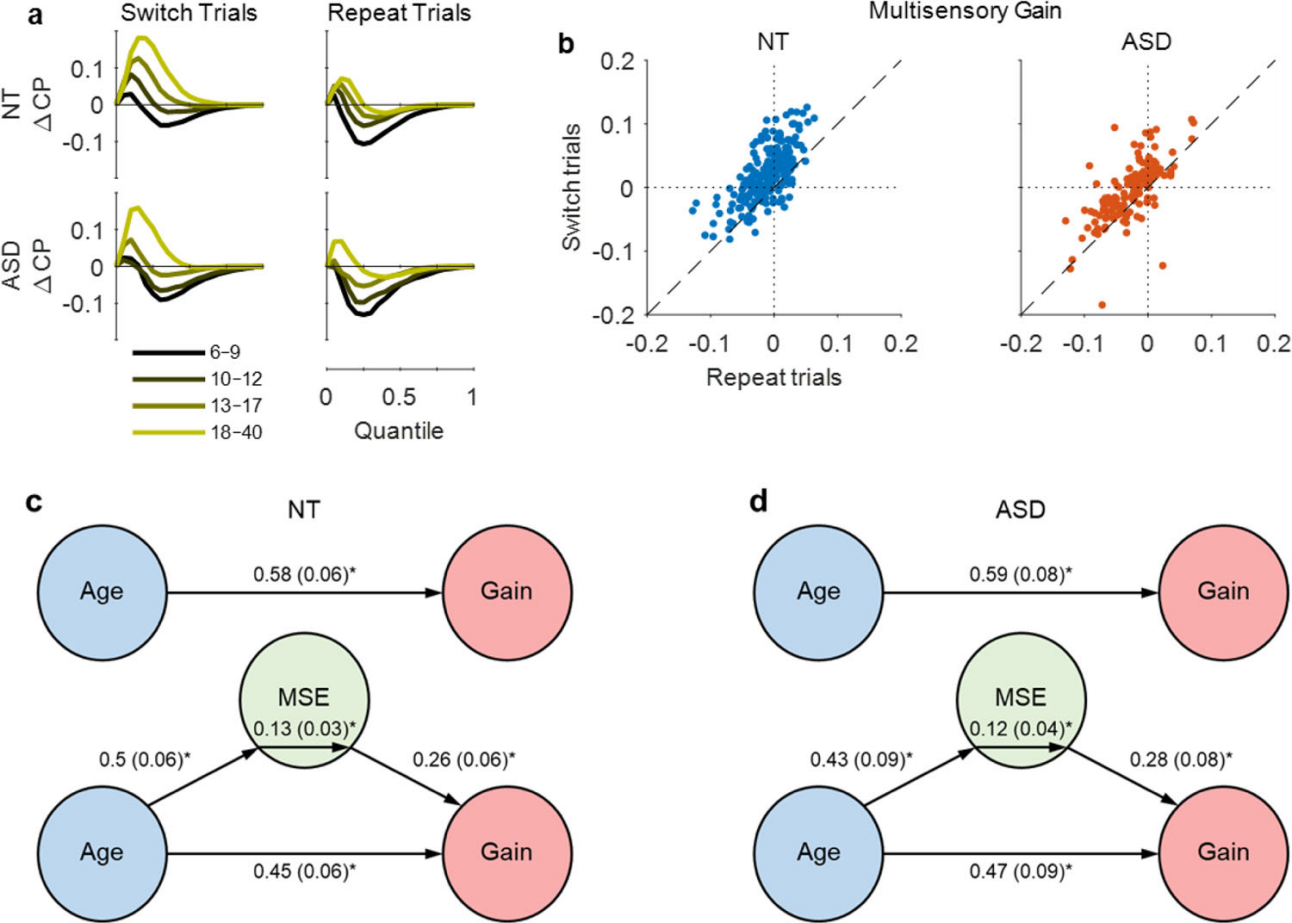
Linking sequential and multisensory effects. **a** Race model violation by diagnosis and age for switch trials (left panel) and repeat trials (right panel). **b** Multisensory gain on switch trials versus repeat trials for NT (left panel) and ASD (right panel) individuals. Each datapoint represents an individual participant. **c**, **d** Mediation model that tested whether modality switch effects (MSEs) mediated the effect of age on multisensory gain. Paths between nodes are labelled with regression coefficients, with SE in parentheses (*p < 0.001, bootstrapped). In both groups, age predicted gain (top path), and predicted MSE controlling for gain (lower left path). The middle coefficients indicate formal mediation effects but the significant direct paths between age and gain controlling for MSE (bottom path) suggest only partial mediation, i.e., MSE did not explain all the shared variance between age and gain.

To examine the relationship between modality switch effects and multisensory gain, we performed a series of partial correlations across participants, controlling for age (see Supplementary Table 3). As one might predict, there was a strong positive correlation between multisensory gain on switch trials and MSEs on unisensory trials (but not on multisensory trials). However, there was no significant correlation between multisensory gain on repeat trials and MSEs on unisensory trials, whereas there was a strong positive correlation with MSEs on multisensory trials (see Supplementary Fig. 6). This pattern, which was identical in both groups, confirms that MSEs on unisensory trials are more likely to contribute to multisensory gain. Figure 8a, b illustrates the impact of switching sensory modality on multisensory facilitation. 87% of NT individuals exhibited a larger multisensory gain on switch trials than on repeat trials (*t*_(224)_ = 15.62, *p* = 0.0002, Hedge’s *g* = 0.84, 95CI [0.73, 0.96], BF_01_ = 4 × 10^−35^), with 83% of individuals with ASD showing the same (*t*_(138)_ = 7.22, *p* = 0.0002, Hedge’s *g* = 0.51, 95CI [0.36, 0.66], BF_01_ = 4 × 10^−9^). Nevertheless, when we submitted RTs from the repeat trials to a race model test, both groups exhibited significant levels of deviation from race model (see Supplementary Table 4).

Having established the relationship between MSEs and multisensory gain, we wished to determine whether the contribution of the former was full or partial. To do this, we submitted the data to a mediation analysis^73^. Specifically, we tested whether MSEs mediated the relationship between participant age and multisensory gain (Fig. 8c, d; see Methods section for further details). First, we established that age was a reliable predictor of both MSE (NT: *β* = 0.5, SE = 0.06, *p* = 2 × 10^−5^; ASD: *β* = 0.43, SE = 0.09, *p* = 0.0001) and multisensory gain (NT: *β* = 0.58, SE = 0.06, *p* = 2 × 10^−5^; ASD: *β* = 0.59, SE = 0.08, *p* = 0.0002), meeting the first two criteria for mediation. MSE affected gain, controlling for age (NT: *β* = 0.26, SE = 0.06, *p* = 5 × 10^−5^; ASD: *β* = 0.28, SE = 0.08, *p* = 0.001) and the mediation effect was significant for both groups (NT: *β* = 0.13, SE = 0.03, *p* = 4 × 10^−5^; ASD: *β* = 0.12, SE = 0.04, *p* = 0.001). However, there was still a significant direct path between age and gain when controlling for MSE (NT: *β* = 0.45, SE = 0.06, *p* = 1 × 10^−5^; ASD: *β* = 0.47, SE = 0.09, *p* = 5 × 10^−5^), indicating that MSE only partially mediated the observed relationship between age and multisensory gain.

## Discussion

Our data suggest that the resolution of multisensory deficits in ASD generalizes to the case of non-social AV stimuli, but that the trajectory is later than that observed in AV speech studies^25,35^. We hypothesized that this delay may be due to lack of environmental exposure to such ecologically-rare stimuli, as this would limit the opportunity to strengthen the necessary excitatory cross-sensory connections, which we know only emerge postnatally with considerable exposure to multisensory experiences^41–44^. Alternatively, delayed resolution of multisensory deficits could result from invocation of neural processes with longer developmental trajectories. Indeed, multisensory gain in NT individuals has been shown to reach full maturity much later for simple AV stimuli^38^ compared to AV speech stimuli^74^. This undoubtedly affects the average age that individuals with ASD will “catch up” to their NT peers.

The disparity in multisensory function trajectories for speech and non-speech stimuli may reflect the fact that multisensory processing occurs across distributed networks and that different stimuli and tasks tap into unique processes with varying trajectories^75^. The task employed in the current study required the speeded detection of simple AV stimuli, without discrimination, identification, or any other higher-order cognitive processing. Integration of such simple AV stimuli likely consists of early cross-sensory activation of visual and auditory cortical regions, enhancing detection of the incoming visual and auditory inputs, respectively^6,76,77^. In contrast, speech identification engages an extensive network of hierarchically-organised brain areas, mapping spectrotemporal representations to phonetic representations, and from there to lexical-semantic representations^78,79^. Moreover, integration of auditory and visual speech cues may act through multiple integrative mechanisms, including early visual activation of auditory cortex, increasing perceptual sensitivity^80^, and later integration of visual speech content (i.e., place and/or manner of articulation), reducing the density of phonemic and lexical neighbourhoods^81,82^. Clearly, task demands and stimuli play a major role in the patterns of multisensory deficits and their resolution that are observed for any given experimental paradigm.

Alternatively, differences in age-related changes in multisensory function could be caused by invocation of neural processes distinct from those under study. Phenomena such as modality switch effects, which contribute significantly to multisensory gain in a bisensory detection task, but not in an AV speech identification task, could prolong the perceived trajectory of multisensory processing. While this is consistent with the fact that age-related changes in MSEs (visual to auditory) extended well into adulthood (Fig. 6b, left), the trajectory of multisensory gain was qualitatively unchanged when the contribution of MSEs was reduced by focusing our analysis on the repeat trials (see Supplementary Fig. 7 and Supplementary Table 4). This, and the results of our mediation analysis, suggest that MSEs are not the sole driving factor behind the disparity in multisensory processing trajectories across the two paradigms.

Human behavioural studies have demonstrated the co-occurrence of multisensory competition and facilitation using RT measures^51^. The existence of a visual dominance in adults, i.e., the Colavita visual dominance effect^72^, means that directing participants to respond to either the auditory or visual component of an AV stimulus can have an inhibitive or facilitative effect on RTs, respectively^52^. However, in the same way that the race model is used as a threshold for detecting facilitative multisensory processing, an upper statistical bound should be used to quantify genuine competitive multisensory procesing^64^. Current neuro-computational perspectives of multisensory development suggest that competition is the default state of integration in the neonatal mammalian brain^45^. Intuitively, a competition scenario would likely favour the most effective sensory modality, which in our case would be either the modality that is most dominant due to an inherent sensory bias (e.g., Colavita effect), or the preceding modality due to prior allocation of attentional resources. To gain insight into the nature of multisensory processing in children and individuals with ASD, we tested two computational models that reflected the above hypothetical scenarios of multisensory competition. We examined fits between the empirical data and model behaviour that were based on a parametric weighting of the race model (facilitation) and each bias model (competition). In children with ASD aged 6–12 years, model fits suggested that the response to an AV stimulus was biased towards the previous sensory modality, potentially due to the presence of competitive processing, whereas in their NT peers, the same models provided only marginal improvements beyond probability summation. This suggests that NT individuals acquire the ability to process multisensory information in a facilitative manner at an earlier age than their ASD peers, who do not show evidence of facilitation until adolescence. This is consistent with the age at which we observe the emergence of multisensory facilitation within each group (see Fig. 2a, b).

Another interesting finding to emerge from our modelling analysis was that NT children aged 6–9 years appear to be biased towards the auditory modality during AV processing, but thereafter are biased towards the visual modality. The same pattern was demonstrated by a follow-up analysis that examined MSE patterns on multisensory trials. These findings are supported by previous studies that have reported an auditory dominance in infants and young children presented with AV stimuli^83,84^, as well as the abovementioned Colavita visual dominance effect commonly reported in adults^72^. Several studies have traced the transition from an auditory to a visual dominance over the course of childhood^85,86^ and, in line with our data, suggest that this sensory reweighting occurs at ~9–10 years of age^86^ (for a meta-analysis, see Hirst, et al.^87^). Sensory reweighting has also been shown to occur ~8–10 years of age for the visual and haptic modalities^49^. Our modelling analysis suggests that the same trend appears to emerge in children with ASD between the ages of 6–12 years, but then reverses once more during adolescence, preferencing the auditory modality in adulthood. Our MSE analysis suggests that a visual dominance exists initially in children with ASD, shifting to an auditory dominance in adulthood. Indeed, a visual dominance has been previously reported in children with ASD^88,89^, but its progression with age has not yet been documented to our knowledge. In support of a visual dominance in ASD, our MSE analysis revealed that children and teenagers with ASD found it easier to switch from auditory to the visual stimuli compared to their NT peers.

Prior work by our lab suggests that the neural processes underlying multisensory integration are impaired in children with autism^36^. Specifically, we found that EEG correlates of integration were weaker (of lower amplitude) and occurred later in the information processing hierarchy. Neural indices of integration over parieto-occipital scalp between 140 and 160 ms were predictive of race model violation in NT children but not in children with ASD. Using the same paradigm, we recorded intracranial electrophysiology in adults with epilepsy and demonstrated that visual stimulation influenced the phase of ongoing oscillations in auditory cortex^77^, and auditory stimulation influenced the phase of ongoing oscillations in visual cortex^76^, such that cross-sensory stimulation appears to prime ancillary sensory cortices to make them more receptive to their primary sensory input. The response to the primary sensory input (e.g., visual stimulation of visual cortex) is then enhanced for multisensory trials^76^, at least in a bisensory detection task such as the current one. Furthermore, neuro-oscillatory phase alignment across the sensorimotor network was significantly enhanced by multisensory stimulation, and was related to the speed of a response^77^.

Phase resetting of ongoing neural oscillations by functionally distinct and distant neuronal ensembles is thought to be fundamental to multisensory integration^90–94^. Impaired cross-sensory phase-resetting, as might be predicted by reduced subcortical and cortical connectivity, would likely result in impaired integrative abilities. In autism, there is evidence for such disrupted connectivity^95,96^, although these findings are mixed and somewhat inconclusive^97^. Nevertheless, disrupted connectivity could in turn lead to impaired cross-sensory phase-resetting and hence contribute to impaired multisensory processing in ASD. As previously mentioned, weaker cross-sensory inhibition might account for reduced MSEs in ASD^65^, possibly as a result of poorer brain connectivity. Alternatively, it may be that cross-sensory connectivity is fully intact in children with ASD, but integration of multisensory information has not yet transitioned from a state of competition, to one of facilitation, as discussed earlier^46,47^. Recent neurocomputational work by our lab has examined the link between such an inhibitory neural architecture and the empirical data present here^53,98^. Establishing the specific neural mechanisms that underlie impaired multisensory behaviour in children with ASD will likely require the use of more advanced neuroimaging techniques.

One of the unexpected findings to emerge from our MSE analysis was the reduced switch costs in adolescents with ASD. This ran contrary to a recent study by Williams et al.^63^ that reported larger switch costs (auditory to visual) in individuals with ASD of approximately the same age. Interestingly, a post hoc analysis of our data that focused on trials with longer ISIs (closer to that of Williams et al.^63^) led to a considerable reduction in mean MSEs but did not yield any evidence of a group difference. While we were unable to detect it empirically due to the limited range of ISIs used in our study (1–3 s), it is possible that there exists an interaction between group and ISI. A possible explanation for this potential interaction points to the so-called “trace theory” which originates from research on MSEs in individuals with schizophrenia^99^. This theory suggests that sensory information leaves traces of residual activity in different neuronal populations, facilitating the processing of subsequent stimuli of the same sensory modality and inhibiting the processing of stimuli of other modalities. Zubin^99^ predicted that these traces attenuate over time but persist longer in individuals with schizophrenia. If such an inhibitory cross-sensory mechanism were weaker in individuals with ASD, but persisted longer over time, it would explain the potential interaction and the inconsistency between our findings and that of Williams et al.^63^. Evidence in support of this theory comes from a recent study that demonstrated that individuals with ASD weight recent stimuli less heavily than NT individuals and that their perception is dominated by longer-term statistics^100^. While reduced cross-sensory inhibition would undoubtedly facilitate processing of subsequent inputs in other sensory systems and thus lead to lower MSEs in ASD, it would also likely result in greater susceptibility to distraction by task/sensory-irrelevant information. This is consistent with previous neurophysiological research by our lab that demonstrated increased susceptibility to distraction by task-irrelevant stimuli in children with ASD^65^. This behavioural deficit was accompanied by a reduced neural suppression of sensory-irrelevant information, as indexed by EEG recordings of alpha-band oscillatory activity. Thus, both behavioural and neurophysiological accounts of multisensory attention in ASD are consistent with a reduction in MSE.

Alternatively, reduced MSEs in ASD could be explained by differences in the ability to make predictions about the sensory environment. While individuals with ASD have been shown to utilise longer-term statistics to make predictions about their sensory environment^100^, other work suggests that they tend to overestimate the volatility of their environment at the expense of learning to build stable predictions^101^. In the current study, stimuli were presented in a random order with equal probability, meaning there was a 66.6% chance of the same unisensory input occurring on the next trial (including the AV condition). Based on these statistics, it is more efficient to predict the reoccurrence of the same signal (or part of it) on the next trial and to direct attention therein. If these statistics are not being actively used to build predictions about the modality of an upcoming stimulus, as may be the case in ASD, then the participant may be less likely to prepare for it and thus less averse to switching sensory modality. This fits well with the notion that individuals with autism rely more on bottom-up than top-down processing^102^.

It is well established that MSEs systematically contribute to multisensory facilitation in a bisensory detection task^60,61,103^. To determine the role of MSEs, we performed separate tests of the race model using switch and repeat trials. While we found that multisensory gain was much greater on switch trials than on repeat trials, there was still evidence of facilitation on repeat trials. However, it is important to consider that in the context of a mixed block design, responses on repeat trials are likely subject to residual switch effects from earlier trials (*n*−2, *n*−3, etc.). Furthermore, if we consider the impact that switching modality has on RTs, a mixed block design could be said to violate the assumption of context invariance. While it is unlikely that it would present the opportunity to change strategy from trial to trial in a top-down manner, it is conceivable that the continuously changing context (from switch to repeat conditions) could invoke disparate processing mechanisms in a bottom-up manner (for a detailed discussion, see Shaw et al.^62^). We also measured the correlation between multisensory gain and MSEs on unisensory and multisensory trials, partialling out the effects of age. There was a strong positive correlation for unisensory (but not multisensory) stimuli, as would be expected if MSEs were to impact multisensory gain systematically. This was followed up with a mediation analysis to determine whether MSEs mediated the observed relationship between age and multisensory gain. This analysis indicated only partial mediation, suggesting that neural processes other than MSEs (e.g., cross-sensory integration) were contributing to the observed multisensory gain.

Another way to examine the contribution of MSEs is to remove the presence of switch trials by using a blocked design. In another study by our lab^62^, we demonstrated that RTs to simple AV stimuli were not faster than race model predictions when the three conditions are presented in entirely separate blocks. Comparing the median RTs between blocked and mixed conditions revealed a slowing of the unisensory but not the multisensory RTs in the mixed condition that could be largely accounted for by increased RTs on switch trials. A similar null result was reported for an AV word detection task that used a blocked continuous speech paradigm^104^. In another study that employed a blocked design, Otto and Mamassian^64^ reported evidence of race model violation, but importantly, presented AV stimuli in background noise which are more likely to recruit integrative mechanisms during bisensory detection^14,18,105,106^. However, it is important to consider the theoretical implications of employing a blocked design. The race model test relies on the assumption of context invariance, because unisensory RTs distributions are used to model multisensory RTs distributions^5,58,107^. By randomly interleaving the conditions, the participant does not know which condition to expect and presumably processes, say, an auditory signal in the same way under unisensory and multisensory conditions. Thus, positive deviations from the race model are assumed to be caused by multisensory integration as opposed to differences in processing strategies. In contrast, when unisensory and multisensory stimuli are presented in separate blocks, there may be opportunity for the subject to employ different processing strategies to optimise task performance. Hence, it is inherently difficult to disentangle contributions of switching and integration when examining the RSE. Violation of the race model likely involves an interplay between integrative and switching processes that carry different weights in different contexts (mixed vs. blocked presentations) and under different stimulus conditions (clean vs. noisy).

We can draw several conclusions from the present study. (1) When assessed using the race model test, multisensory processing in individuals with ASD has “normalized” to neurotypical levels by adulthood. (2) Computational modelling suggested that multisensory processing in children with ASD takes longer to transition from the default mode of competition to facilitation. (3) Different age-related patterns in sensory dominance indicate fundamental alterations in how the nervous system of children with ASD responds in a dynamic multisensory environment. (4) The behavioural cost of switching from auditory to visual stimuli is smaller in teenagers with ASD, possibly due to altered cross-sensory inhibition or reduced influence of short-term statistics. The current findings also make clear that there is significant work ahead of us before we fully understand the neural processes that contribute to age-related changes in multisensory function and how it differs in children with ASD. Complicating this endeavour, we must begin to understand how such empirical evidence collected in high-functioning individual with ASD translates to those most affected by the disorder and how future work can strive to obtain meaningful data from low-functioning individuals with ASD. Here we set the stage for detailed characterisation of these processes and their interactions, to in turn understand potential roadblocks to the typical age-related changes in multisensory processing in ASD, and some of the factors that might contribute to sensory reactivity in both high- and low-functioning individuals within this group.

## Methods

The present study is based on reanalysis of a large body of data collected as part of several previously published studies^36,38,108^, as well as unpublished data from more than 200 additional participants.

### Participants

A total of 411 individuals participated in the experiment. The data of 47 participants (11.4% of the total sample, 34 ASD) were excluded from all analyses based on the following criteria: (1) they did not fall within the desired age range of 6–40 years (*n* = 9), (2) their performance IQ was below 80 or not recorded (*n* = 24), (3) their detection accuracy was <3 SDs below the sample’s mean (<65%, *n* = 6), (4) they had an excessive number of false alarms (>65%, *n* = 4), (5) they had a disproportionate number of hits on visual trials (excessive eye-closure) or on audio trials (not listening; <50% of other modality, *n* = 3), (6) the ISIs used were not within the desired range of 1–3 seconds (*n* = 1), or (7) they had less than 20 RTs per condition (*n* = 1; this can bias median RT estimates^109,110^ as well as race model estimates^111^). Of the remaining 364 participants, 225 met criteria for NT (age range: 6–36 years; 115 females) and 139 had a diagnosis of ASD (age range: 6–39 years; 34 females). For analysis purposes, age was treated either as a continuous variable or participants were cross-sectioned into four age groups: children (6–9 years), pre-adolescents (10–12 years), adolescents (13–17 years), adults (18–40 years). Mean age was not statistically different between NT and ASD participants in any of the four age groups (statistics reported in Supplementary Table 1). Participant demographics are presented in Table 1.

Individuals were excluded from participating in the experiment if they had a history of seizures or head trauma, or a known genetic disorder. Additionally, NT participants were excluded if they had a history of psychiatric, educational, attentional or other developmental difficulties (as assessed by a history questionnaire), a biological first-degree relative with a known developmental disorder, or if they or their legal guardians endorsed six or more items of inattention or hyperactivity on a DSM-IV checklist for attention deficit disorder. For the vast majority of participants, diagnoses of ASD were obtained by a trained clinical psychologist using the Autism Diagnostic Interview-Revised^112^ and the Autism Diagnostic Observation Schedule (ADOS)^113^. Diagnoses of the remaining individuals were made by a licensed clinical psychologist external to this study using the Diagnostic Criteria for Autistic Disorder from the DSM-IV-TR^114^. For more details regarding sub-phenotyping, medication and ethnic demographics, please refer to previous studies^36,108^.

Intelligence quotients for performance (PIQ), verbal (VIQ) and full-scale (FSIQ) were assessed in 65% of NT participants and 97% of ASD participants using the Wechsler Abbreviated Scales of Intelligence (WASI)^115^. We found no evidence to suggest that mean PIQ was statistically different between NT and ASD participants in any of the four age groups (statistics described in Supplementary Table 1). To ensure rigorous between-group comparisons, individuals within each subgroup were matched for sex, age and PIQ using a *k*-nearest neighbour search. The descriptive statistics for each of the subgroups are summarised in Table 1. Participants were formally screened for normal or corrected-to-normal vision using a Snellen eye test chart and audiometric threshold evaluation confirmed that all participants had within-normal-limits hearing. All procedures were approved by the institutional review boards of the City College of New York, Albert Einstein College of Medicine, and University of Rochester School of Medicine and Dentistry. All participants or legal guardians of participants provided written informed consent in accordance with the tenets of the 1964 Declaration of Helsinki.

### Stimuli and procedure

The stimulus materials were identical to those described previously^38^. In brief, visual (V) stimuli consisted of a red disc (diameter: 3.2 cm; duration: 60 ms), located 0.4 cm above a central fixation crosshair on a black background. The disc subtended visual angles of 1.5° vertically and horizontally and the bottom of the disc subtended 0.9° vertically above the crosshair. Auditory (A) stimuli consisted of a 1-kHz pure tone, sampled at 44.1 kHz (duration: 60 ms; rise/fall time: 5 ms; intensity: 75 dB SPL). Audiovisual (AV) stimuli consisted of the combined simultaneous pairing of the auditory and visual stimuli described above (see Supplementary Fig. 1).

Participants performed a speeded bisensory detection task on a computer and were seated 122 cm from the visual display in a dimly lit, sound-attenuated booth. RTs were recorded during the simultaneous recording of electrophysiological (EEG) data, however, the EEG data are not reported in this study (please refer to previous studies^36,38,108^). To reduce predictability, the stimuli were presented in a completely randomised order with equal probability and the interstimulus interval (ISI) was randomly jittered between 1000 and 3000 ms according to a uniform, square-wave distribution. The task did not involve any catch trials or background noise/distractors. Stimulus presentation was controlled using Presentation® software (Neurobehavioral Systems, Inc., Berkeley, CA). Auditory stimuli were delivered binaurally at an intensity of 75 dB SPL via a single, centrally located loudspeaker (JBL Duet Speaker System, Harman Multimedia). Visual stimuli were presented at a resolution of 1280 × 1024 pixels on a 17-inch Flat Panel LCD monitor (Dell Ultrasharp 1704FTP). The auditory and visual stimuli were presented in close spatial proximity, with the speaker placed atop the monitor and aligned vertically to the visual stimulus. Participants were instructed to press a button on a response pad (Logitech Wingman Precision Gamepad) with their right thumb as soon as they perceived any of the three stimuli. Analogue triggers indicating the latencies of stimulus onsets and button presses were sent to the acquisition PC via Presentation® and stored digitally at a sampling rate of 512 Hz in a separate channel of the EEG data file using ActiView software (BioSemi™, Amsterdam, The Netherlands). Stimuli were presented in blocks of ~100 trials and participants typically completed 6–10 blocks in total.

### Data analysis

To account for false alarms and excessive button pressing, detection accuracy was quantified by the harmonic mean of precision and recall, i.e., F_1_ score^116^. Response times were measured relative to the onset time of the preceding stimulus and analysed in MATLAB (The MathWorks, Inc., Natick, MA). Responses were excluded from all analyses if there was more than one response within a given trial (double-presses), they occurred within the first 3 trials of a block (considered training) or the preceding ISI was not between 1000 and 3000 ms (due to system errors). An outlier correction procedure was performed before the main RT analyses. First, RTs that did not fall within 100 and 2000 ms post-stimulus were removed. On average, fast outliers (<100 ms, considered anticipatory responses) made up 0.7% (±0.9) of trials and slow outliers (>2000 ms, considered misses) made up 0.4% (±0.6) of trials. Second, RTs outside the middle 95th percentile (2.5–97.5) of their respective conditions were removed. While not all RTs outside of this range are necessarily outliers, those within this range are most likely to come from the cognitive processes under consideration^117^. This approach minimises the impact of outliers with only negligible truncation biases^118^ and captures the range of RTs at an individual-participant level, an important factor when dealing with significant inter-subject variability.

Data analysis was conducted on the whole RT distribution by splitting it into discrete quantiles^67,68^. RTs were organised into 20 linearly-spaced quantiles between the 2.5 and 97.5 cutoffs used for outlier correction. Because outlier correction was performed separately for each condition, the lowest 2.5 and highest 97.5 percentiles were used for all three conditions to maintain the relationship between them. Cumulative distribution functions (CDFs) were obtained by calculating the cumulative probability of RTs occurring below time *t* of each quantile given a signal *X*, $P\left({{{{{{\rm{RT}}}}}}}_{{{{{{\rm{X}}}}}}}\le t|{{{{{\rm{X}}}}}}\right)$. The resulting CDFs can be represented as a function of time *t* or quantiles in the unit interval [0, 1]. Here, we use quantiles for consistency across different analyses and in keeping with previous work^119^. We chose to quantify the CDFs as cumulative probabilities (the “vertical” method), as opposed to taking the additional step to interpolate them to reaction times (the “horizontal” method). This is particularly important when dealing with a highly heterogeneous population, as is the case here, because a speed-up indexed by the proportion of faster RTs can reveal relative effects that could otherwise be obscured when measured in absolute time (seconds). Note, this approach does not require there to be an equal number of RTs in each condition^120^.

### Race model analysis

To obtain quantitative predictions of statistical facilitation, we used Raab’s independent race model^4^. Let $P\left({{{{{{\rm{RT}}}}}}}_{{{{{{\rm{A}}}}}}}\le t|{{{{{\rm{AV}}}}}}\right)$ and $P\left({{{{{{\rm{RT}}}}}}}_{{{{{{\rm{V}}}}}}}\le t|{{{{{\rm{AV}}}}}}\right)$ represent the CDFs of the A and V components of an AV stimulus, respectively. Assuming the RT distributions of the A and V components overlap, the probability of either triggering a response can be represented using probability summation. To solve this analytically, we need to make two assumptions: (1) RTs to the A and V components of the AV signal follow the same distributions as the RTs to the unisensory A and V signals, such that $P\left({{{{{{\rm{RT}}}}}}}_{{{{{{\rm{A}}}}}}}\le t|{{{{{\rm{AV}}}}}}\right)=P\left({{{{{{\rm{RT}}}}}}}_{{{{{{\rm{A}}}}}}}\le t|{{{{{\rm{A}}}}}}\right)$ and $P\left({{{{{{\rm{RT}}}}}}}_{{{{{{\rm{V}}}}}}}\le t|{{{{{\rm{AV}}}}}}\right)=P\left({{{{{{\rm{RT}}}}}}}_{{{{{{\rm{V}}}}}}}\le t|{{{{{\rm{V}}}}}}\right)$, i.e., context invariance^107,121,122^; (2) RTs to the A and V components of the AV signal are statistically independent, such that their joint probability $P\left({{{{{{\rm{RT}}}}}}}_{{{{{{\rm{A}}}}}}\cap {{{{{\rm{V}}}}}}}\le t|{{{{{\rm{AV}}}}}}\right)$ can be calculated by the product of $P\left({{{{{{\rm{RT}}}}}}}_{{{{{{\rm{A}}}}}}}\le t|{{{{{\rm{A}}}}}}\right)$ and $P\left({{{{{{\rm{RT}}}}}}}_{{{{{{\rm{V}}}}}}}\le t|{{{{{\rm{V}}}}}}\right)$^123^. Simplifying $P\left({{{{{{\rm{RT}}}}}}}_{{{{{{\rm{A}}}}}}\cup {{{{{\rm{V}}}}}}}\le t|{{{{{\rm{AV}}}}}}\right)$ to *F*_A∪V_ (*t*), $P\left({{{{{{\rm{RT}}}}}}}_{{{{{{\rm{A}}}}}}}\le t|{{{{{\rm{A}}}}}}\right)$ to *F*_A_ (*t*) and $P\left({{{{{{\rm{RT}}}}}}}_{{{{{{\rm{V}}}}}}}\le t|{{{{{\rm{V}}}}}}\right)$ to *F*_V_ (*t*), the race model can be represented as:

1 $$\begin{array}{c}{F}_{{{{{{\rm{A}}}}}}\cup {{{{{\rm{V}}}}}}}\left(t\right)={F}_{{{{{{\rm{A}}}}}}}\left(t\right)+{F}_{{{{{{\rm{V}}}}}}}\left(t\right)-{F}_{{{{{{\rm{A}}}}}}}\left(t\right)\cdot {F}_{{{{{{\rm{V}}}}}}}\left(t\right)\end{array}$$

Note, the joint probability term is often omitted from Eq. 1 to produce an upper bound of statistical facilitation known as Miller’s bound or the race model inequality^5^, as the assumption of channel independence is poorly motivated; it is likely that responses to signals on different sensory channels compete for resources^5,58,124–126^. Assuming that the allocation of such resources is partially determined by the modality of the previous trial^5^, we separated the unisensory RTs by preceding sensory modality (A, V, AV) and computed individual race models per condition before averaging them:

2 $$\begin{array}{c}{\bar{F}}_{{{{{{\rm{A}}}}}}\cup {{{{{\rm{V}}}}}}}\left(t\right)=\frac{1}{3}\mathop{\sum }\limits_{m=1}^{3}{F}_{{{{{{\rm{A}}}}}}\cup {{{{{\rm{V}}}}}}}\left(m,t\right)\end{array}$$

where *m* is the preceding modality. This method captured some of the dependency between RTs to signals on different channels, resulting in an estimate of statistical facilitation that was less conservative on 90% of quantiles over the 364 participants (two-tailed permutation tests with *t*_max_ correction).

Multisensory benefits were quantified by the area between the CDFs in the multisensory condition and the most effective unisensory condition^54^. First, we computed the multisensory benefit predicted by the race model (Fig. 1b, left):

3 $$\begin{array}{c}{{{\mbox{benefit}}}}_{{{\mbox{pred}}}}={\int }_{0}^{1}{\bar{F}}_{{{{{{\rm{A}}}}}}\cup {{{{{\rm{V}}}}}}}\left(t\right)-{\max }\left[{F}_{{{{{{\rm{A}}}}}}}\left(t\right),{F}_{{{{{{\rm{V}}}}}}}\left(t\right)\right]{dt}\end{array}$$

where the integral is taken over every quantile *t* from 0 to 1. ${\max }\left[{F}_{{{{{{\rm{A}}}}}}}\left(t\right),{F}_{{{{{{\rm{V}}}}}}}\left(t\right)\right]$ represents a lower bound on statistical facilitation, known as Grice’s bound^127^. Similarly, we computed empirical benefits based on the observed multisensory RTs (Fig. 1b, right):

4 $$\begin{array}{c}{{{\mbox{benefit}}}}_{{{\mbox{emp}}}}={\int }_{0}^{1}{F}_{{{{{{\rm{AV}}}}}}}\left(t\right)-{\max }\left[{F}_{{{{{{\rm{A}}}}}}}\left(t\right),{F}_{{{{{{\rm{V}}}}}}}\left(t\right)\right]{dt}\end{array}$$

To determine whether the empirical multisensory benefits exceeded the statistical facilitation predicted by the race model, we computed the difference between the CDFs of the multisensory condition and the race model at every quantile^6^. Positive deviations indicate quantiles where multisensory RTs were faster than predicted, i.e., facilitation. To obtain an overall index of multisensory gain, we calculated the area under the curve (AUC) by taking the integral over every quantile as before (Fig. 3a):

5 $${{{{{\mathrm{gain}}}}}}={\int }_{0}^{1}{F}_{{{{{{\rm{AV}}}}}}}\left(t\right)-{{\bar F}}_{{{{{{\rm{A}}}}}}\cup {{{{{\rm{V}}}}}}}\left(t\right){dt}$$

While it is common practice to interpret only the positive AUC as an index of multisensory gain^12,128,129^, Eq. 6 is equal to the sum of the positive and negative AUC^130,131^. This is mathematically equivalent to the difference between predicted and empirical benefits and represents the overall multisensory gain across the whole RT distribution. Qualitatively, this is equivalent to using only the positive AUC^132^, because the positive and negative AUCs are inversely proportional (see Fig. 3b). Moreover, many younger participants in this study did not exhibit a multisensory benefit that exceeded statistical facilitation, rendering a statistical analysis based on only the positive AUC less powerful. All race model analyses were conducted using the RaceModel (v1.0) toolbox (https://github.com/mickcrosse/RaceModel).

### Modelling multisensory competition

To determine whether subject behaviour reflected multisensory processing of a facilitative or competitive nature, we attempted to model the logical coupling between parallel decision processes for both cases. Facilitation would likely follow the predictions of the race model as it exactly matches the task demands of a bisensory detection task, i.e., a logical disjunction^59^. Competition, on the other hand, would likely follow the predictions of the stronger sensory modality because of cross-sensory inhibitory mechanisms^53^, regardless of inherent processing speeds on either channel. Such biased channel coupling could manifest as a result of two potential scenarios: (1) the dominant sensory modality would generally prevail due to an inherent biological preference, thus triggering the response or (2) the modality of the previous trial would generally prevail due to pre-allocated attentional resources, thus triggering the response. The first scenario was modelled with a bias towards either the auditory modality (Model 1A) or the visual modality (Model 1V) as follows:

6 $${F}_{1b}\left(t\right)=\frac{1}{3}\mathop{\sum }\limits_{m=1}^{3}{F}_{b}\left(m,t\right)$$

where *m* is the preceding modality (A, V, AV) and *b* is the modality that the system is biased towards (A or V). The second scenario was modelled with a bias towards the previous modality, except when the previous trial was an AV stimulus, where it was biased towards either the auditory modality (Model 2A) or the visual modality (Model 2V):

7 $$\begin{array}{c}{F}_{2b}\left(t\right)=\frac{1}{3}\left({F}_{{{{{{\rm{A}}}}}}}\left({{{{{\rm{A}}}}}},t\right)+{F}_{{{{{{\rm{V}}}}}}}\left({{{{{\rm{V}}}}}},t\right)+{F}_{b}\left({{{{{\rm{AV}}}}}},t\right)\right)\end{array}$$

The models were used to obtain alternative measures of predicted benefits and assessed based on how accurately they predicted empirical benefits. To examine potential age-related transitions in multisensory processing, we parametrically varied the probability of such processing being facilitative (race model) or competitive (bias model) as follows:

8 $$\begin{array}{c}{{{\mbox{benefit}}}}_{{ib}}={\int }_{0}^{1}\left(1-p\right){\bar{F}}_{{{{{{\rm{A}}}}}}\cup {{{{{\rm{V}}}}}}}\left(t\right)+p{F}_{{ib}}\left(t\right)-{\max }\left[{F}_{{{{{{\rm{A}}}}}}}\left(t\right),{F}_{{{{{{\rm{V}}}}}}}\left(t\right)\right]{dt},{{{{{\rm{for}}}}}}\,p=0,0.25,\ldots 1\end{array}$$

where *F*_*ib*_ (*t*) is the bias model and *p* is the probability of it triggering a response. When *p* = 0, processing is purely facilitative (race model), and when *p* = 1, processing is purely competitive (bias model). In addition, Model 1 was used to examine age-related changes in sensory dominance which are likely to have a significant impact during competitive multisensory processing.

### Quantifying modality switch effects

To examine modality switch effects (MSEs), RTs were separated into trials preceded by the same modality (repeat trials) and those preceded by a different modality (switch trials). Unisensory trials preceded by multisensory trials (AV→A, AV→V) were excluded from this analysis as they were considered neither switches nor repeats (repeat trials: A→A, V→V, AV→AV; switch trials: V→A, A→V, V→AV, A→AV). Separate CDFs were obtained for switch and repeat trials within each condition. Trials belonging to the two multisensory switch conditions (A→AV, V→AV) were pooled to produce one multisensory switch condition (V/A→AV). MSEs were quantified by the area between the CDFs of the switch and repeat trials:

9 $$\begin{array}{c}{{\mbox{MSE}}}={\int }_{0}^{1}{F}_{{{{{{\rm{repeat}}}}}}}\left(t\right)-{F}_{{{{{{\rm{switch}}}}}}}\left(t\right){dt}\end{array}$$

To examine the impact of switching sensory modality on the observed multisensory gain, separate tests of the race model were performed for switch and repeat trials.

### Modelling channel dependency and RT variability

Seminal work by Otto and Mamassian^64^ has demonstrated that the basic architecture of race models can be used to accurately predict empirical multisensory RT data by including two additional free parameters to account for (1) the additional variability or noise *η* typically observed in empirical multisensory RTs relative to standard race model predictions, and (2) the correlation *ρ* between processing speeds on different sensory channels. Conceptually, Miller’s and Grice’s bounds assume a perfect negative and positive correlation respectively, whereas Raab’s model assumes zero correlation (i.e., independence). Otto’s model on the other hand makes no such assumptions, allowing the correlation parameter *ρ* to vary in a way that optimises the model’s fit to the empirical data. Using the RSE-box^133^ (v1.0) toolbox (https://github.com/tomotto/RSE-box), we examined the values of *ρ* and *η* that optimised the model fit for each participant. Gaussian functions were fit to the reciprocal of the unisensory RT distributions via the LATER model approach^134^, which assumes that the reciprocals of the RT distributions are normally distributed with mean *μ* and SD *σ* (see Fig. 7b). These parameters were then used to generate the probability density function of the maximum distribution ${f}_{A\cup V}(x)={f}_{A}(-x)+{f}_{V}(-x)$ (refs. ^64,135^).

### Statistics and reproducibility

A linear mixed-effects model was used to determine which parameters influenced RTs, fit using the maximum likelihood criterion. Single-trial RTs were the continuous numeric dependent variable. Diagnosis was a contrast-coded fixed factor (NT, ASD), age was a continuous numeric fixed factor (6–40 years), and condition was a multi-level nominal fixed factor (AV, A, V). Subjects were included as a random factor, along with by-subject slope adjustments for condition^66^. ISI was included as another random factor, as well as preceding modality with slope adjustments for condition. Subsequent analyses employing standard linear models coded fixed effects as above.

A mediation analysis^136^ was conducted using the M3 Mediation (v1.0) toolbox (https://github.com/canlab/MediationToolbox) to establish whether the relationship between participants’ age and multisensory gain was mediated by a direct effect of age on MSE. For this analysis, MSEs were averaged across the two unisensory conditions (V→A, A→V), as we hypothesized that it was a slowing of unisensory RTs that was the cause of the observed RSE. We constructed a three-variable mediation model with age as the causal variable, gain as the outcome variable and MSE as the mediating variable (see Fig. 8c). All 3 variables were z-scored prior to conducting the analysis. For MSE to be considered a mediator, the following criteria must be met based on three separate regressions: (1) the causal variable must affect the outcome, (2) the causal variable must affect the mediator, and 3) the mediator must affect the outcome but the causal variable must either no longer affect the outcome (full mediation) or at least weaken the effect (partial mediation). Significance and SE of the associated path coefficients were bootstrapped (10,000 samples) and adjusted using the bias-corrected and accelerated percentile method^73^.

Significance testing was conducted via nonparametric permutation tests (10,000 permutations) using the PERMUTOOLS (v1.0) toolbox (https://github.com/mickcrosse/PERMUTOOLS). Multivariate tests were adjusted to control for family-wise error rate using the *t*_*max*_ correction method^137–139^. This method has been shown to control for Type 1 error at a desired level when performing tests of the race model at multiple quantiles^69^. Moving mean analyses were corrected for multiple comparisons using the false discovery rate (FDR) control procedure^140^. Bias-corrected effect sizes (i.e., Hedge’s *g*^141^) were calculated using the MES (v1.0) toolbox^141^ (https://github.com/hhentschke/measures-of-effect-size-toolbox). All confidence intervals were bootstrapped (10,000 samples) at the 95% confidence level and adjusted using the bias-corrected and accelerated percentile method^142^. Bayes factor (BF_01_) analyses were conducted using the Bayes Factor (v2.0) toolbox (https://github.com/klabhub/bayesFactor) to test for the absence of an effect by quantifying the relative likelihood of the data under the null versus the alternative hypothesis. Effect sizes were assumed to follow a Cauchy prior distribution with a scale parameter of 1 and were interpreted using the standard convention^139^.

### Reporting summary

Further information on research design is available in the Nature Research Reporting Summary linked to this article.

## Supplementary information

Supplementary Information

Reporting Summary

## Data Availability

The data underlying figures and corresponding demographic information that support the findings of the study are available to download in anonymised form from Figshare (https://figshare.com/s/d349bd507d419db8077f).

## Code availability

The custom MATLAB scripts used to run the main and supplementary analyses of the study are available to download from GitHub (https://github.com/mickcrosse/AVSRT-Autism). Dependencies for conducting the main modelling and statistical analyses have been packaged by the authors as the abovementioned RaceModel and PERMUTOOLS toolboxes, respectively, and are available to download from GitHub.
