# Supplementary material for: Resolution of impaired multisensory processing in autism and the cost of switching sensory modality
Source: Commun Biol. 2022 Jun 30;5:601. doi: 10.1038/s42003-022-03519-1 (PMC9246932; doi:10.1038/s42003-022-03519-1)
Supplement: Supplementary file 1 — Supplementary Information [file 42003_2022_3519_MOESM1_ESM.pdf]

Supplementary Information

Resolution of Impaired Multisensory Processing in Autism and the Cost of Switching Sensory Modality

Michael J. **Crosse**[1,2,3,*], John J. **Foxe**[1,2,4], Katy **Tarrit**[4], Edward G. **Freedman**[4] and Sophie **Molholm**[1,2,4,*]

[1]The Cognitive Neurophysiology Laboratory, Department of Pediatrics, Albert Einstein College of Medicine, Bronx, NY, USA. [2]The Dominick P. Purpura Department of Neuroscience, Rose F. Kennedy Intellectual and Developmental Disabilities Research Center, Albert Einstein College of Medicine, Bronx, NY, USA. [3]Trinity Centre for Biomedical Engineering, Department of Mechanical, Manufacturing & Biomedical Engineering, Trinity College Dublin, Dublin, Ireland. [4]The Cognitive Neurophysiology Laboratory, Del Monte Institute for Neuroscience, Department of Neuroscience, University of Rochester School of Medicine and Dentistry, Rochester, NY, USA. [*]e-mail: crossemi@tcd.ie; sophie.molholm@einsteinmed.org

Supplementary Figures

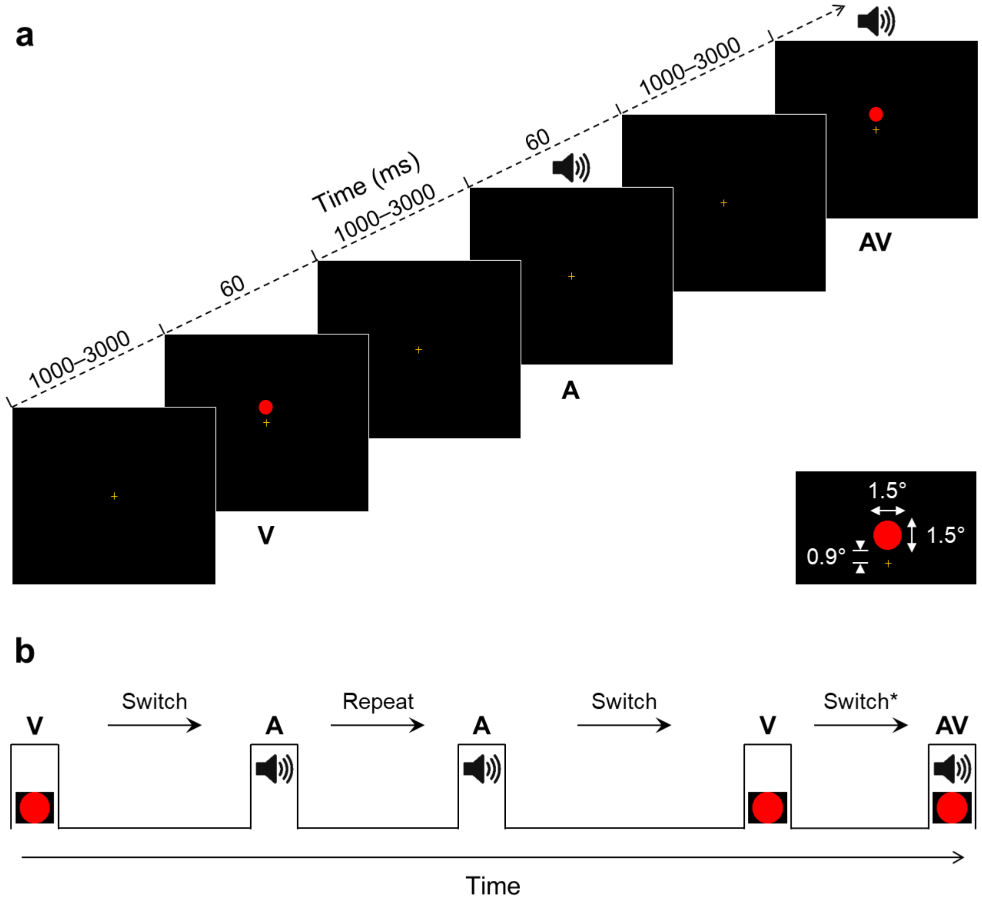

Supplementary Fig. 1 | Bisensory detection task. a, Auditory (A), visual (V) and audiovisual (AV) stimuli (60-ms duration) were presented in a randomized order every 1000–3000 ms. Participants responded to each stimulus as fast as possible with a button press. **b**, Stimuli were categorized as either switch or repeat trials based on the modality of the preceding stimulus (repeat trials: AV→AV, A→A, V→V; switch trials: V→AV*, A→AV*, A→V, V→A). Asterisks indicate trials that are only partial switches. Trials AV→A and AV→V were excluded from the analysis as they were considered neither switches nor repeats.

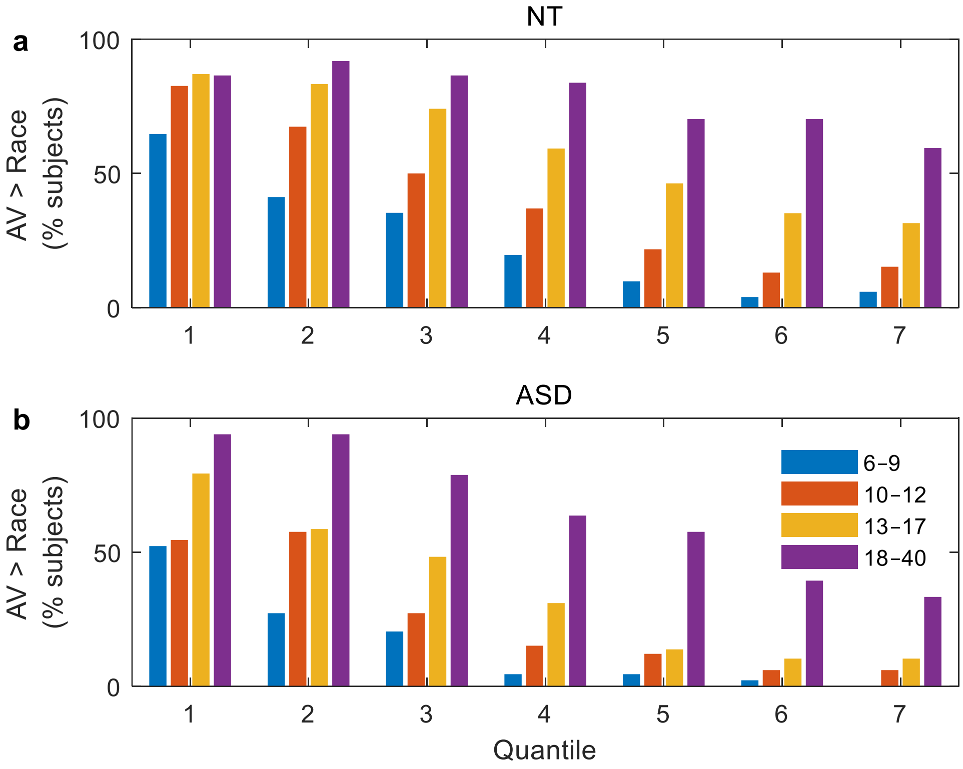

Supplementary Fig. 2 | Multisensory facilitation. a, **b**, Percentage of subjects showing evidence of multisensory facilitation (indexed by positive deviations from the race model) at each of the first 7 quantiles of their RT distributions for NT and ASD participants, respectively. Age group is indicated by colour.

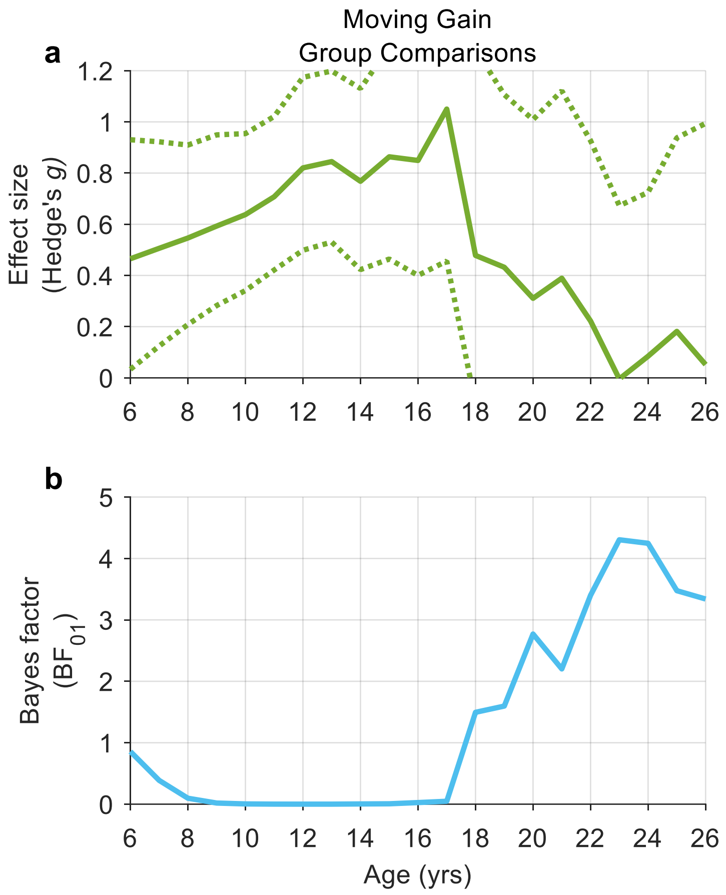

Supplementary Fig. 3 | Moving gain group comparisons. a, Effect size (Hedge's *g*) with 95% CIs (dotted trace) based on the group comparisons (NT vs. ASD) for the moving mean analysis of multisensory gain (AV − race model). See Fig. 3f of main article for moving mean analysis. Each group comparison was conducted using a 7-year moving window in increments of 1 year from 6–26 years. Participants were sex, age and IQ-matched within each 7-year window. **b**, Bayes factor (BF_{01}) to test for evidence of the absence of a group effect (i.e., resolution). BF_{01} values greater than 3 provide moderate evidence in favour of the absence of an effect.

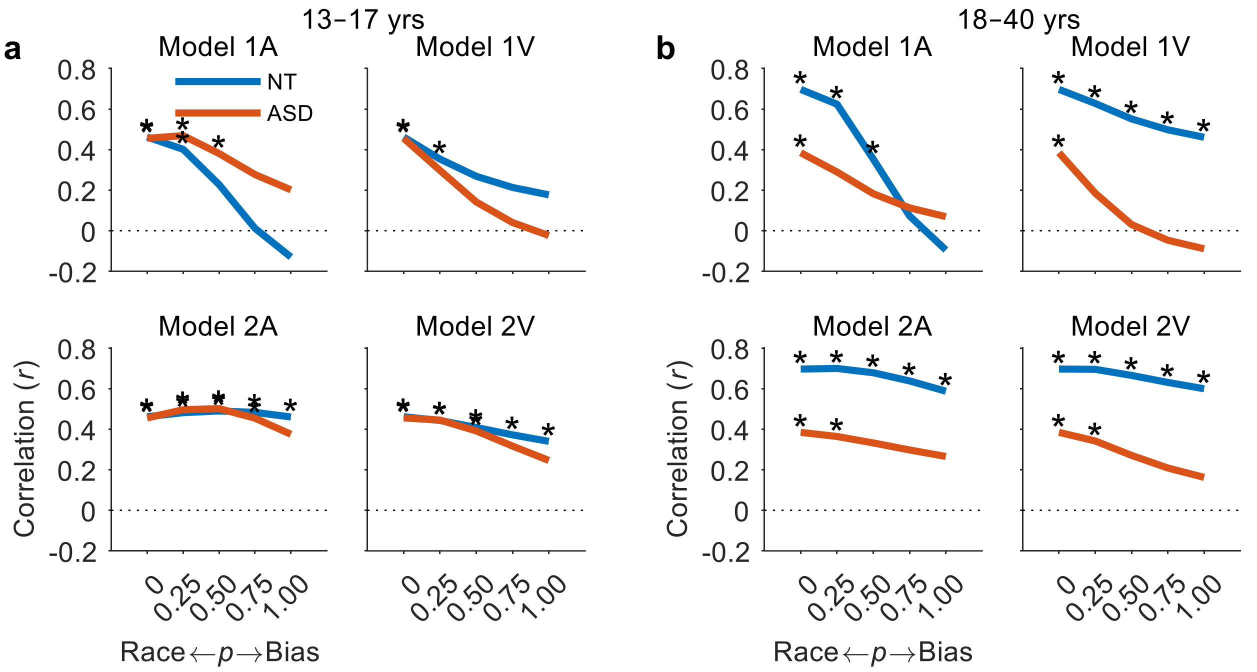

Supplementary Fig. 4 | Modelling multisensory behaviour in teenagers and adults. a, **b**, Hypothetical models of multisensory competition were tested. Model 1A was biased towards the auditory modality and Model 1V towards the visual modality. Model 2A was biased towards the preceding modality and the A modality when preceded by an AV trial, and Model 2V was biased towards the preceding modality and the V modality when preceded by an AV trial. The probability p of multisensory processing being facilitative (race model) or competitive (bias model) was parametrically varied between 0 and 1 in increments of 0.25. The ability of the models to predict empirical benefits was assessed within each age group based on the Pearson correlation coefficient. Data presented are the two older age groups. See Fig. 4c,d of main article for the two younger age groups.

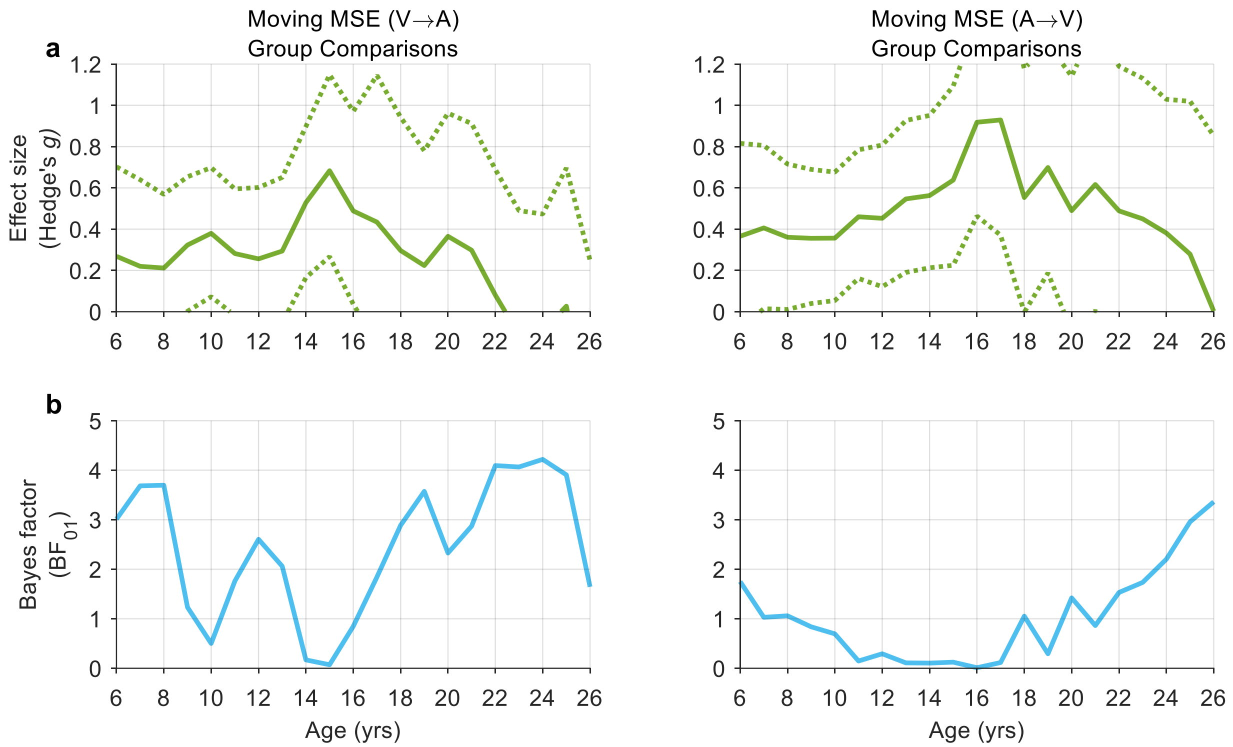

Supplementary Fig. 5 | Moving MSE group comparisons. a, Effect size (Hedge's *g*) with 95% CIs (dotted trace) based on the group comparisons (NT vs. ASD) for the moving mean analysis of MSE for visual to auditory (left panel) and auditory to visual (right panel) switches. See Fig. 6b of main article for moving mean analysis. Each group comparison was conducted using a 7-year moving window in increments of 1 year from 6–26 years. Participants were sex, age and IQ-matched within each 7-year window. **b**, Bayes factor (BF_{01}) to test for evidence of the absence of a group effect (i.e., resolution). BF_{01} values greater than 3 provide moderate evidence in favour of the absence of an effect.

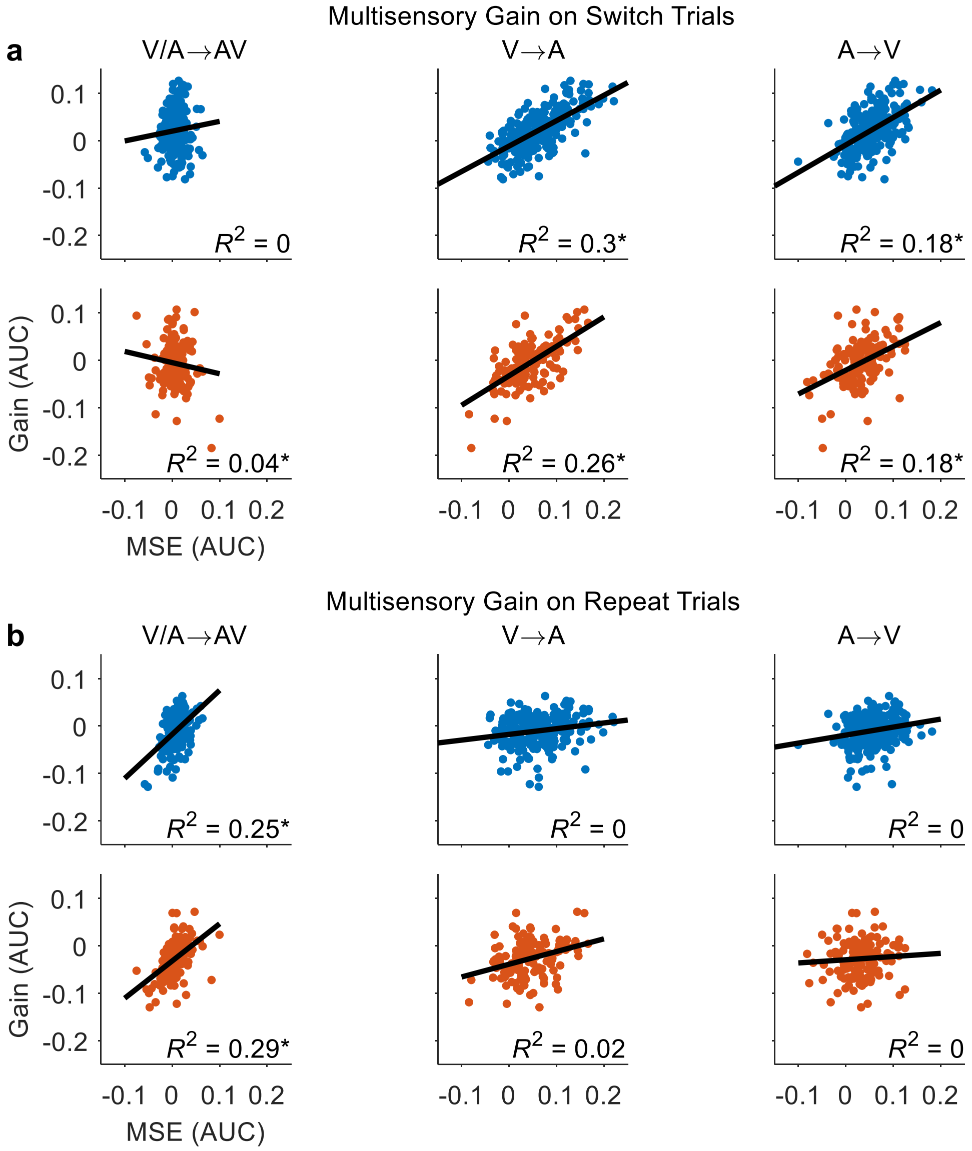

Supplementary Fig. 6 | Relationship between sequential and multisensory effects. a, **b**, Correlation between modality switch effects (MSE) and multisensory gain by condition and diagnosis (blue = NT, red = ASD) for switch trials and repeat trials, respectively. Each datapoint represents the area under the curve (AUC) for an individual participant. Explained variance (R^2) of the regression lines and its significance are displayed in the bottom right corner of each panel (*$p < 0.05$; partial correlation controlling for age).

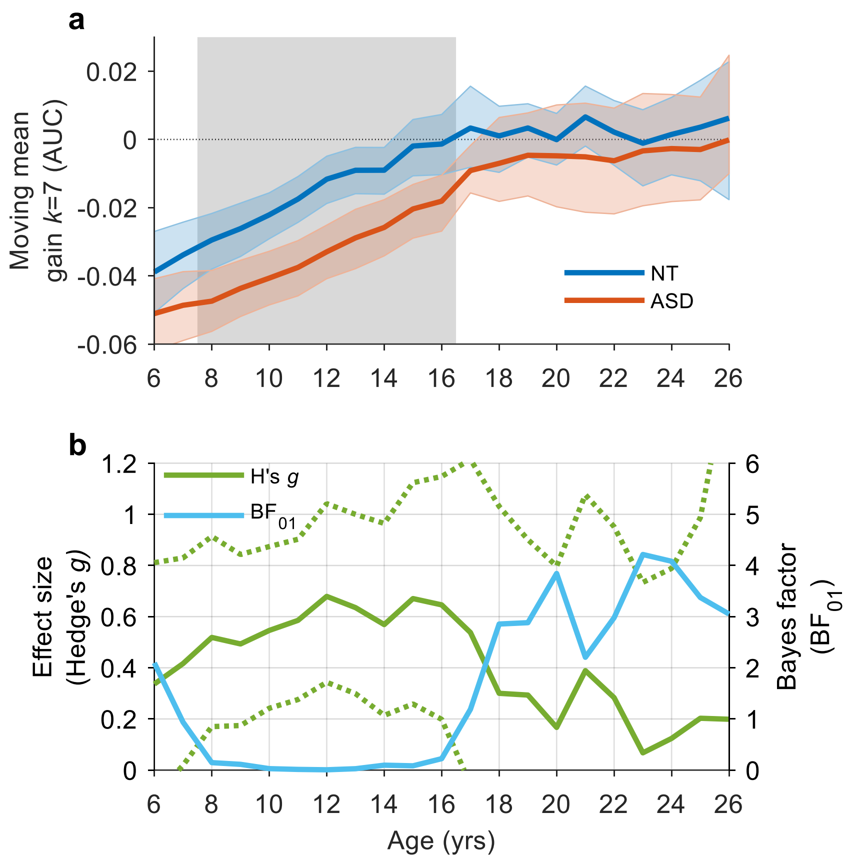

Supplementary Fig. 7 | Multisensory gain based on repeat trials. a, Mean multisensory gain calculated with a moving window k of 7 years in increments of 1 year from 6–26 years for NT (blue trace) and ASD (red trace) participants. Participants were sex, age and IQ-matched within each 7-year window. Coloured error bounds indicate 95% CIs (bootstrapped). Gray shaded regions indicate significant group differences ($p < 0.05$, two-tailed permutation tests, FDR corrected). **b**, Effect size (Hedge's *g*; green trace) with 95% CIs (dotted trace) and Bayes factor (BF_{01}; light blue trace) based on the group comparisons for the moving mean analysis of multisensory gain. BF_{01} values greater than 3 provide moderate evidence in favour of the absence of an effect.

Supplementary Tables

Supplementary Table 1 | Group comparisons of age, F1 score and intelligence quotients for each age bin. Values shown indicate the significance of the two-tailed permutation tests (*p*) and corrected effect sizes (Hedge's *g*).

	NT vs. ASD			
	6–9 yrs	10–12 yrs	13–17 yrs	18–40 yrs
Age	p = 0.776, g = -0.05	p = 0.664, g = 0.10	p = 0.294, g = 0.24	p = 0.536, g = 0.13
F1	p = 0.001, g = 0.69	p = 0.000, g = 0.90	p = 0.017, g = 0.57	p = 0.000, g = 0.80
PIQ	p = 0.972, g = -0.00	p = 0.309, g = 0.23	p = 0.288, g = -0.25	p = 0.655, g = 0.15
VIQ	p = 0.000, g = 0.99	p = 0.002, g = 0.78	p = 0.000, g = 0.84	p = 0.096, g = 0.60
FSIQ	p = 0.003, g = 0.65	p = 0.006, g = 0.65	p = 0.057, g = 0.44	p = 0.154, g = 0.51

Supplementary Table 2 | Test statistics comparing CDFs of multisensory RTs with the race model (the first 7 quantiles are shown for brevity, but all 20 quantiles were tested). Values shown indicate effect sizes (Hedge's *g*) and 95% CIs (bootstrapped) in brackets. Asterisks indicate significant positive deviation from the race model, i.e., facilitation ($p < 0.05$, right-tailed permutation tests, t_{max} corrected).

	NT				ASD			
Q	6–9 yrs	10–12 yrs	13–17 yrs	18–40 yrs	6–9 yrs	10–12 yrs	13–17 yrs	18–40 yrs
1	0.18[0.1,0.3]*	0.44[0.2,0.7]*	0.54[0.4,0.9]*	0.83[0.6,1.1]*	0.12[-0.0,0.3]	0.08[-0.1,0.3]	0.28[0.1,0.6]*	0.77[0.5,1.2]*
2	-0.06[-0.2,0.1]	0.21[0.1,0.4]*	0.41[0.3,0.6]*	0.73[0.6,0.9]*	-0.14[-0.2,-0.0]	-0.05[-0.2,0.1]	0.07[-0.1,0.3]	0.51[0.3,0.8]*
3	-0.21[-0.3,-0.1]	0.03[-0.1,0.2]	0.29[0.2,0.4]*	0.59[0.4,0.8]*	-0.29[-0.4,-0.2]	-0.18[-0.3,-0.0]	-0.08[-0.3,0.1]	0.34[0.2,0.6]*
4	-0.35[-0.5,-0.2]	-0.11[-0.2,-0.0]	0.17[0.1,0.3]*	0.44[0.3,0.6]*	-0.48[-0.7,-0.3]	-0.34[-0.6,-0.2]	-0.27[-0.5,-0.1]	0.22[0.0,0.5]
5	-0.53[-0.8,-0.4]	-0.27[-0.4,-0.2]	0.03[-0.1,0.2]	0.31[0.2,0.4]*	-0.66[-1.1,-0.5]	-0.49[-0.8,-0.3]	-0.52[-0.9,-0.3]	0.11[-0.1,0.3]
6	-0.80[-1.1,-0.6]	-0.43[-0.6,-0.3]	-0.09[-0.2,0.0]	0.21[0.1,0.3]*	-0.89[-1.4,-0.6]	-0.67[-1.1,-0.5]	-0.73[-1.3,-0.4]	0.00[-0.2,0.2]
7	-1.00[-1.4,-0.8]	-0.55[-0.8,-0.4]	-0.20[-0.4,-0.1]	0.12[-0.0,0.2]	-1.12[-1.7,-0.8]	-0.73[-1.3,-0.5]	-0.92[-1.5,-0.6]	-0.10[-0.3,0.0]

Supplementary Table 3 | Partial correlations between multisensory gain and MSEs, controlling for age. Multisensory gain was computed separately for switch trials (left columns) and repeat trials (right columns). Values shown indicate coefficients of determination (R^2) and significance of the correlation (p).

	Multisensory Gain on Switch Trials			Multisensory Gain on Repeat Trials		
	V/A→AV	V→A	A→V	V/A→AV	V→A	A→V
NT	$R^2 = 0.001$	$R^2 = 0.3$	$R^2 = 0.18$	$R^2 = 0.25$	$R^2 = 9\times10^{-7}$	$R^2 = 0.003$
	$p = 0.7$	$p = 3\times10^{-19}$	$p = 3\times10^{-11}$	$p = 1\times10^{-15}$	$p = 0.99$	$p = 0.42$
ASD	$R^2 = 0.04$	$R^2 = 0.26$	$R^2 = 0.18$	$R^2 = 0.29$	$R^2 = 0.02$	$R^2 = 0.0001$
	$p = 0.02$	$p = 2\times10^{-10}$	$p = 2\times10^{-7}$	$p = 1\times10^{-11}$	$p = 0.12$	$p = 0.89$

Supplementary Table 4 | Test statistics comparing CDFs of multisensory RTs with the race model using only repeat trials (the first 7 quantiles are shown for brevity, but all 20 quantiles were tested). Values shown indicate effect sizes (Hedge's g) and 95% CIs (bootstrapped) in brackets. Asterisks indicate significant positive deviation from the race model, i.e., facilitation ($p < 0.05$, right-tailed permutation tests, t_{max} corrected).

	NT				ASD			
Q	6–9	10–12	13–17	18–40	6–9	10–12	13–17	18–40
1	0.28[0.1,0.5]*	0.48[0.3,0.8]*	0.48[0.3,0.7]*	0.73[0.5,1.0]*	0.10[-0.1,0.3]	-0.02[-0.3,0.3]	0.14[-0.1,0.4]	0.79[0.6,1.1]*
2	-0.13[-0.3,0.0]	0.14[-0.0,0.3]	0.27[0.1,0.4]*	0.50[0.4,0.7]*	-0.24[-0.4,-0.1]	-0.08[-0.3,0.1]	-0.08[-0.3,0.1]	0.35[0.2,0.6]*
3	-0.27[-0.4,-0.1]	-0.09[-0.2,0.1]	0.11[-0.0,0.2]	0.38[0.2,0.5]*	-0.44[-0.6,-0.3]	-0.28[-0.5,-0.1]	-0.15[-0.4,0.1]	0.15[-0.1,0.4]
4	-0.45[-0.7,-0.3]	-0.25[-0.4,-0.1]	-0.02[-0.2,0.1]	0.20[0.1,0.3]*	-0.60[-0.9,-0.4]	-0.45[-0.8,-0.3]	-0.35[-0.7,-0.1]	0.04[-0.2,0.3]
5	-0.65[-1.0,-0.5]	-0.42[-0.6,-0.3]	-0.17[-0.3,-0.0]	-0.01[-0.2,0.1]	-0.74[-1.2,-0.5]	-0.60[-1.0,-0.4]	-0.57[-1.0,-0.3]	-0.07[-0.3,0.1]
6	-0.88[-1.3,-0.7]	-0.57[-0.8,-0.4]	-0.24[-0.4,-0.1]	-0.13[-0.3,0.0]	-0.99[-1.5,-0.7]	-0.70[-1.1,-0.5]	-0.81[-1.3,-0.5]	-0.15[-0.4,0.0]
7	-1.01[-1.4,-0.8]	-0.69[-1.0,-0.5]	-0.38[-0.7,-0.2]	-0.28[-0.5,-0.1]	-1.11[-1.7,-0.7]	-0.71[-1.1,-0.5]	-0.91[-1.4,-0.5]	-0.25[-0.5,-0.1]
