# Supplementary material for: Resolution of impaired multisensory processing in autism and the cost of switching sensory modality
Source: Commun Biol. 2022 Jun 30;5:601. doi: 10.1038/s42003-022-03519-1 (PMC9246932; doi:10.1038/s42003-022-03519-1)
Supplement: Supplementary file 2 — Reporting Summary [file 42003_2022_3519_MOESM2_ESM.pdf]

nature portfolio

Corresponding author(s): Michael Crosse, Sophie Molholm

Last updated by author(s): May 4, 2022

# Reporting Summary

Nature Portfolio wishes to improve the reproducibility of the work that we publish. This form provides structure for consistency and transparency in reporting. For further information on Nature Portfolio policies, see our Editorial Policies and the Editorial Policy Checklist.

## Statistics

For all statistical analyses, confirm that the following items are present in the figure legend, table legend, main text, or Methods section.

| n/a | Confirmed | |
|---|---|---|
| ☐ | ☒ | The exact sample size (*n*) for each experimental group/condition, given as a discrete number and unit of measurement |
| ☐ | ☒ | A statement on whether measurements were taken from distinct samples or whether the same sample was measured repeatedly |
| ☐ | ☒ | The statistical test(s) used AND whether they are one- or two-sided<br>*Only common tests should be described solely by name; describe more complex techniques in the Methods section.* |
| ☐ | ☒ | A description of all covariates tested |
| ☐ | ☒ | A description of any assumptions or corrections, such as tests of normality and adjustment for multiple comparisons |
| ☐ | ☒ | A full description of the statistical parameters including central tendency (e.g. means) or other basic estimates (e.g. regression coefficient) AND variation (e.g. standard deviation) or associated estimates of uncertainty (e.g. confidence intervals) |
| ☐ | ☒ | For null hypothesis testing, the test statistic (e.g. *F*, *t*, *r*) with confidence intervals, effect sizes, degrees of freedom and *P* value noted<br>*Give P values as exact values whenever suitable.* |
| ☐ | ☒ | For Bayesian analysis, information on the choice of priors and Markov chain Monte Carlo settings |
| ☒ | ☐ | For hierarchical and complex designs, identification of the appropriate level for tests and full reporting of outcomes |
| ☐ | ☒ | Estimates of effect sizes (e.g. Cohen's *d*, Pearson's *r*), indicating how they were calculated |

*Our web collection on statistics for biologists contains articles on many of the points above.*

## Software and code

Policy information about availability of computer code

| | |
|---|---|
| Data collection | Data was collected using Presentation® software (Neurobehavioral Systems, Inc., Berkeley, CA). |
| Data analysis | Data analysis was performed using custom MATLAB scripts (https://github.com/mickcrosse/AVSRT-Autism). Dependencies: modelling analyses were conducted in RaceModel (v1.0) and RSE-box (v1.0); frequentist statistics were conducted in PERMUTOOLS (v1.0) and MES (v1.0); Bayesian statistics were conducted in Bayes Factor (v.20); mediation analyses were conducted in M3 Mediation Toolbox (v1.0). |

For manuscripts utilizing custom algorithms or software that are central to the research but not yet described in published literature, software must be made available to editors and reviewers. We strongly encourage code deposition in a community repository (e.g. GitHub). See the Nature Portfolio guidelines for submitting code & software for further information.

## Data

Policy information about availability of data

All manuscripts must include a data availability statement. This statement should provide the following information, where applicable:

- Accession codes, unique identifiers, or web links for publicly available datasets
- A description of any restrictions on data availability
- For clinical datasets or third party data, please ensure that the statement adheres to our policy

The data and corresponding demographic information that support the findings of the study are available to download in anonymised form from Figshare (https://figshare.com/s/d349bd507d419db8077f).

# Field-specific reporting

Please select the one below that is the best fit for your research. If you are not sure, read the appropriate sections before making your selection.

☒ Life sciences ☐ Behavioural & social sciences ☐ Ecological, evolutionary & environmental sciences

For a reference copy of the document with all sections, see nature.com/documents/nr-reporting-summary-flat.pdf

# Life sciences study design

All studies must disclose on these points even when the disclosure is negative.

| | |
|---|---|
| Sample size | The study has a large sample size due to ongoing data collection efforts over a 10-year period. Sample sizes for each analysis were determined by the smaller of the two cross-sectional groups being compared, with the other group being sex, age and IQ-matched. |
| Data exclusions | 47 of the 411 participants (34 ASD) were excluded from all analyses based on the following criteria: 1) they did not fall within the desired age range of 6–40 years (n = 9), 2) their performance IQ was below 80 or not recorded (n = 24), 3) their detection accuracy was less than 3 SDs below the sample's mean (<65%, n = 6), 4) they had an excessive number of false alarms (>65%, n = 4), 5) they had a disproportionate number of hits on visual trials (excessive eye-closure) or on audio trials (not listening; <50% of other modality, n = 3), 6) the interstimulus intervals used were not within the desired range of 1–3 seconds (n = 1), or 7) they had less than 20 RTs per condition (n = 1).<br>Responses were excluded from all analyses if there was more than one response within a given trial (double-presses), they occurred within the first 3 trials of a block (considered training) or the preceding ISI was not between 1000–3000 ms (due to system errors). RTs that did not fall within 100–2000 ms post-stimulus were removed. On average, fast outliers (<100 ms, considered anticipatory responses) made up 0.7% (±0.9) of trials and slow outliers (>2000 ms, considered misses) made up 0.4% (±0.6) of trials. RTs outside the middle 95th percentile (2.5–97.5) of their respective conditions were removed. |
| Replication | Previous findings by our group (Brandwein et al., 2011, 2013) were successfully replicated in a larger sample. A number of our analyses were also designed to test the same central hypothesis and all of these analyses supported the same conclusions. We attempted to replicate a previous finding by Williams, et al. (2013) but did not. However, our results supported other findings within the present study and the discrepancy between the two studies can be explained by differences in the experimental design which we describe in the manuscript. |
| Randomization | For analysis purposes, age was treated either as a continuous variable or participants were cross-sectioned into four age groups: children (6–9 years), pre-adolescents (10–12 years), adolescents (13–17 years), adults (18–40 years). To ensure rigorous between-group comparisons, individuals within each subgroup were matched for sex, age and PIQ using a k-nearest neighbour search. The descriptive statistics for each of the subgroups are summarized in Table 1. For the moving mean analyses, a window of 7 years was applied in increments of 1 year between the ages of 6 and 26 years. Controls were sex, age and IQ-matched to ASD individuals within each 7-year window. |
| Blinding | It was not possible to blind investigators to group allocation during data collection as participants were either assigned to neurotypical (NT) or autism spectrum disorder (ASD) groups, and either child, pre-adolescent, adolescent or adult groups. |

# Reporting for specific materials, systems and methods

We require information from authors about some types of materials, experimental systems and methods used in many studies. Here, indicate whether each material, system or method listed is relevant to your study. If you are not sure if a list item applies to your research, read the appropriate section before selecting a response.

## Materials & experimental systems

| n/a | Involved in the study | |
|---|---|---|
| ☒ | ☐ | Antibodies |
| ☒ | ☐ | Eukaryotic cell lines |
| ☒ | ☐ | Palaeontology and archaeology |
| ☒ | ☐ | Animals and other organisms |
| ☐ | ☒ | Human research participants |
| ☒ | ☐ | Clinical data |
| ☒ | ☐ | Dual use research of concern |

## Methods

| n/a | Involved in the study | |
|---|---|---|
| ☒ | ☐ | ChIP-seq |
| ☒ | ☐ | Flow cytometry |
| ☒ | ☐ | MRI-based neuroimaging |

## Human research participants

Policy information about studies involving human research participants

| | |
|---|---|
| Population characteristics | Following participant rejection, 225 met criteria for NT (age range: 6–36 years; 115 females) and 139 had a diagnosis of ASD (age range: 6–39 years; 34 females). Participant demographics are presented in Table 1. |
| Recruitment | Participant recruitment was conducted through the Human Clinical Phenotyping and Recruitment Cores of the Intellectual & Developmental Disabilities Research (IDDRC) at the Albert Einstein College of Medicine and the University of Rochester, referrals from clinicians (primarily at the Albert Einstein College of Medicine), advertising, and at community health fairs. |

Neurotypical children and adolescents were recruited from a local junior high school and from a community sample obtained through friends and acquaintances of colleagues and college students. Neurotypical adults were recruited through the The City College of New York's Psychology research subject pool and from a community sample.

| | |
|---|---|
| Ethics oversight | All procedures were approved by the institutional review boards of The City College of New York, Albert Einstein College of Medicine, and University of Rochester School of Medicine and Dentistry. |

Note that full information on the approval of the study protocol must also be provided in the manuscript.
